# A longitudinal study of rotenone-induced Parkinsonism in mice

**DOI:** 10.1016/j.isci.2026.117996

**Published:** 2026-10-23

**Authors:** Prince Joshi, Fan Fan, Xuelin Lou

**Affiliations:** 1 Department of Cell Biology, Neurobiology and Anatomy, Medical College of Wisconsin, Milwaukee, WI 53226, USA

**Keywords:** Parkinson’s disease, pesticides, hydroxylase, mitochondria, non-motor deficit, dopaminergic neurodegeneration, neuroplasticity, SNc neurons

## Abstract

Parkinson’s disease (PD) is a progressive neurodegenerative disorder characterized by the loss of dopaminergic neurons in the substantia nigra. Environmental toxins such as rotenone are widely used to model PD pathology and evaluate new drugs in PD; however, the long-term consequences of toxin exposure remain poorly understood. Here, we performed a longitudinal mouse study to examine the onset, progression, and long-term outcomes of rotenone-induced PD-like phenotypes. A 21-day regimen of rotenone (2.5 mg/kg/day, intraperitoneal) produced robust Parkinsonism-like phenotypes by week 4, including impaired locomotion, anxiety-like behaviors, and deficits in motor coordination and learning, accompanied by reduced tyrosine hydroxylase (TH) expression and loss of TH^+^ neurons in the substantia nigra pars compacta. Unexpectedly, these behavioral and neuropathological deficits gradually resolved over time. After 12 months of toxin cessation, rotenone-treated mice exhibited behavioral performance and TH expression levels comparable to untreated controls. These findings reveal a previously underappreciated capacity for adaptive resilience within the nigrostriatal dopaminergic system following toxic injury.

## Highlights

• Chronic high-dose rotenone exposure induces Parkinsonism-like behaviors
• Rotenone reduces SNc TH^+^ neurons by ∼40% during acute toxicity
• Rotenone-induced Parkinsonism-like behaviors are resolved by 12 months
• PD-like cellular pathology in the SNc is resolved by 12 months

## Graphical abstract

**Figure:**
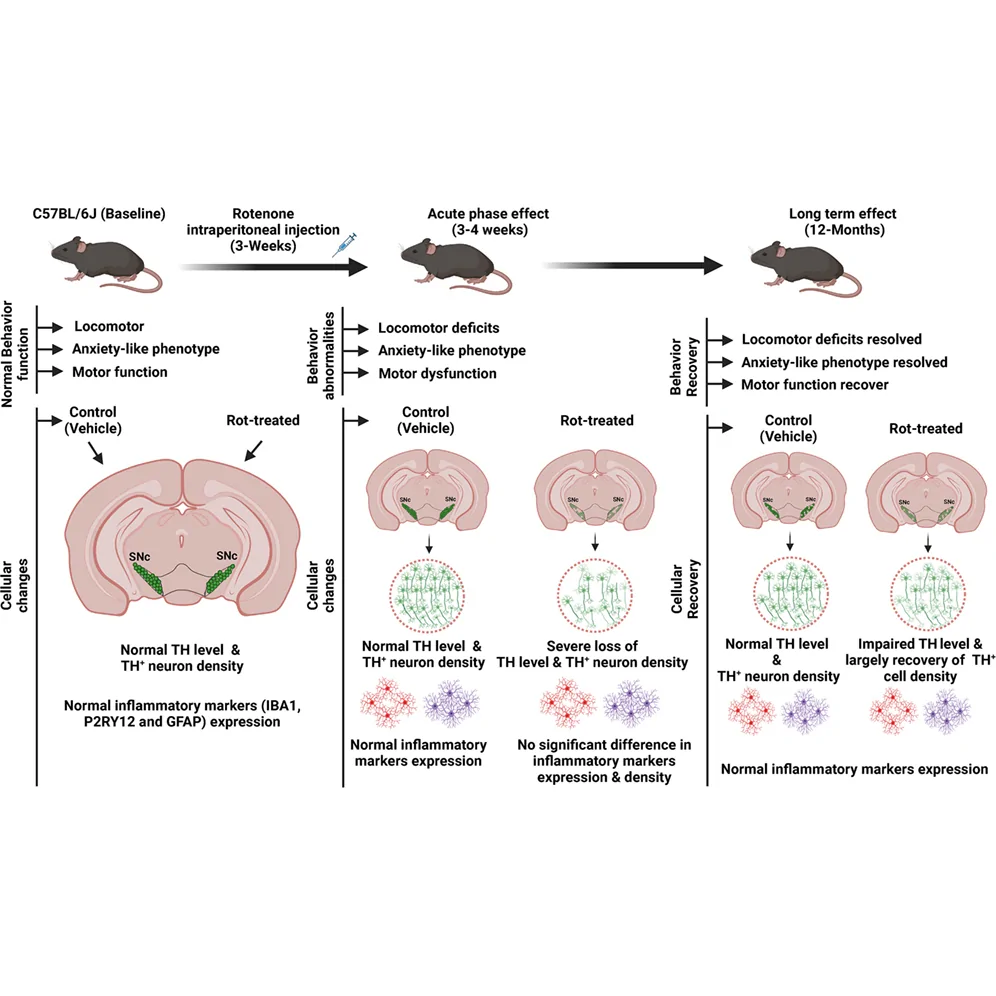

## Teaser

Toxicology; Neuroscience; Cell biology

## Introduction

Parkinson’s disease (PD) is the second most common neurodegenerative disease, but current treatments are limited to symptomatic relief only, and none of them halt or slow disease progression. Clinically, PD exhibits cardinal motor symptoms, including akinesia, bradykinesia, tremor, rigidity, and postural instability, as well as prominent non-motor symptoms such as anxiety, constipation, hyposmia, and sleep disturbance.^1^^,^^2^ These manifestations arise primarily from the progressive loss of dopaminergic (DA) neurons in the substantia nigra pars compacta (SNc) and consequent depletion of dopamine in the caudate putamen. Despite decades of research, PD pathogenesis remains incompletely understood; it may involve a complex interplay between genetic susceptibility and environmental factors converging on key processes such as misfolded α-synuclein, overwhelming metabolism, and chronic inflammation.^2^ Across both sporadic and familial forms of PD, mitochondrial dysfunction has emerged as a central player.^3^^,^^4^ Impaired mitochondrial complex I activity, elevated oxidative stress, and bioenergetic failure are consistently observed in animal models and postmortem PD patient brains. In line with this, exposure to pesticides containing mitochondrial toxins—most notably rotenone and paraquat—has been associated with an increased risk of PD,^5^^,^^6^^,^^7^ but a definitive causal relationship remains uncertain.^5^^,^^6^ Addressing causality requires longitudinal studies that are not feasible in human populations, underscoring the importance of well-controlled animal models.

Rotenone, a widely used pesticide and potent mitochondrial complex I inhibitor, provided early experimental support for the mitochondrial hypothesis of PD. Chronic rotenone infusion in rats via jugular vein cannula for 1–5 weeks can induce selective SNc degeneration, synuclein aggregation, and hypokinesia.^8^ Since this seminal work, rotenone has been increasingly used to model PD-like pathology and to evaluate candidate PD drugs.^9^^,^^10^^,^^11^ It offers distinct advantage as compared with other commonly used toxins, such as 6-hydroxydopamine (6-OHDA), N-methyl-4-phenyl-1,2,3,6-tetrahydropyridine (MPTP), and its metabolite (1-methyl-4-phenylpyridinium [MPP^+^]). The latter toxins rely on dopamine transporter-dependent uptake to induce selective DA lesions, effectively modeling late-stage PD within hours or days of administration,^12^^,^^13^^,^^14^ and thus limiting their ability to capture progressive neurodegeneration or α-synuclein aggregation. In contrast, rotenone exposure better mimics the insidious process onset of PD and reproduces more disease-relevant processes, including mitochondrial impairment,^15^ nigrostriatal DA loss,^8^^,^^16^^,^^17^ α-synuclein inclusions akin to Lewy bodies,^8^^,^^16^^,^^18^ and neuroinflammation.^19^^,^^20^^,^^21^ Notably, transient rotenone exposure in rats has been reported to induce progressive postural instability and delayed α-synuclein aggregation after 9 months of exposure.^18^

While rotenone rat models have been well established for decades,^8^^,^^9^ mouse rotenone models have gained attention only more recently, with different routes of exposure reported.^11^^,^^22^^,^^23^ These models exhibit substantial variability depending on experimental conditions, such as exposure route, age, and sex, with males producing more robust phenotypes than females.^11^^,^^24^^,^^25^ Compared with rat models, outcomes in mice are less consistent; even identical oral exposure paradigms have yielded conflicting results.^16^^,^^26^ Although these rotenone rodent models are valuable *in vivo* systems for interrogating PD pathogenesis and therapeutic strategies, most studies have focused on short-term outcomes, leaving the long-term consequences of rotenone exposure unexplored.

To address this knowledge gap, we systematically evaluated the effectiveness and long-term changes of PD-like behaviors and associated neuropathology induced by a chronic systemic rotenone exposure—3 weeks of intraperitoneal (i.p.) injection. We show that rotenone exposure induces robust Parkinsonism during the acute phase—shortly after exposure cessation. Unexpectedly, however, both behavioral deficits and DA pathology are resolved and become indistinguishable from the control group by 12 months. These findings reveal a previously underappreciated capacity for adaptive compensation and resilience of the DA system *in vivo*, with potential implications for better understanding PD progression and intervention.

## Results

### Chronic systemic rotenone exposure at high doses impairs mouse locomotion during the acute toxicity phase

The age-matched male mice were treated with rotenone and vehicle in parallel for 21 days, with their behavior changes monitored. Before experiments, rotenone was first dissolved in 100% DMSO and then diluted in medium-chain triglyceride Miglyol 812 and injected intraperitoneally (2.5 mg/kg bodyweight per day). Mice receiving rotenone were referred to as the PD model; mice receiving only vehicle in parallel were used as controls. Open field tests (Figure 1A) were performed before the treatments and within the 4^th^ week of treatments, referred to as the acute phase. The results demonstrated a strong impairment in locomotion in rotenone-treated mice (Figure 1). These mice exhibited a significant reduction of total travel distance (Figure 1B), movement speed (Figure S1A), and mobile time (Figure S1E) and an increase in immobile time and freezing time (Figures S1D and S1F) (*n* = 12 mice for the vehicle group; *n* = 15 mice for the PD model group, two-tailed Welch’s *t* test). Thus, systemic rotenone i.p. exposure impairs mouse locomotor function at the acute phase, mimicking motor deficits in human PD.

**Figure 1:**
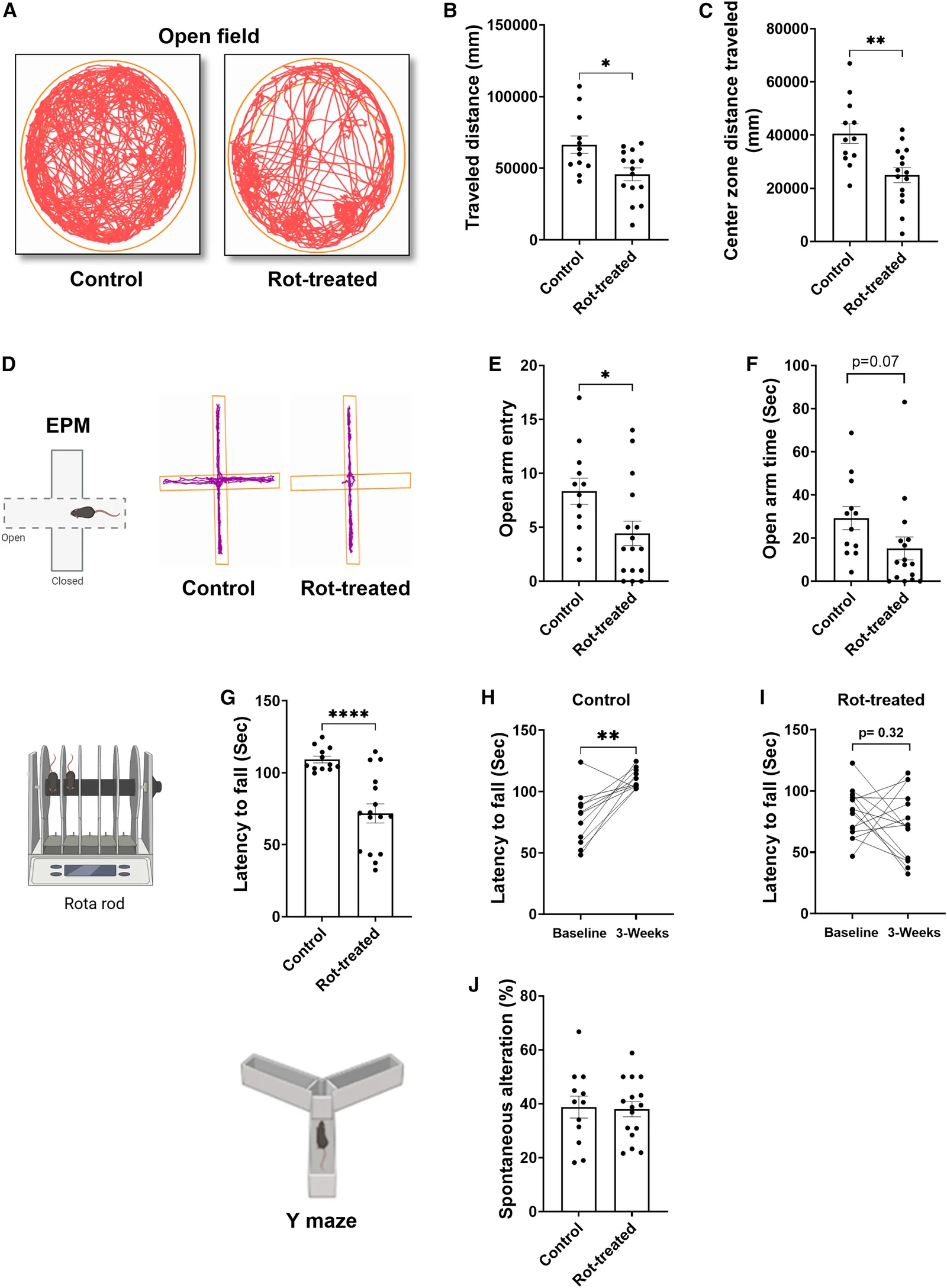
Intraperitoneal rotenone exposure induces locomotor deficits, anxiety-like behaviors, and impaired motor function and motor learning during the acute phase (A) Representative movement tracks in the open field test from a control and a Rot-treated mouse after 3 weeks of treatment. (B and C) Quantification of total distance traveled and center-zone distance traveled. (D) Representative mouse trajectories in the elevated plus maze (EPM). (E and F) Quantification of open-arm entries and time. (G) Latency to fall in the rotarod test. (H and I) Rotarod performance changes between baseline and after treatment completion in control (H) and Rot-treated (I) mice. (J) Spatial working memory assessed by the Y-maze test. Data are presented as mean ± SEM, with individual points representing single animals. Statistical analyses were performed using two-tailed Welch’s *t* tests, except for rotarod performance changes (H and I), which were analyzed using two-tailed paired *t* tests. Sample sizes: open field, control *n* = 12, Rot-treated *n* = 15; EPM, rotarod, and Y-maze, control *n* = 12, Rot-treated *n* = 16. Statistical significance is indicated as ∗*p* < 0.05, ∗∗*p <* 0.01, and ∗∗∗∗*p <* 0.0001.

### Rotenone exposure induces anxiety-like behavior within 4 weeks

In the open field tests described earlier, we observed a significant reduction of travel distance selectively in the center (but not outer) zones (Figures 1C and S1B), a typical sign of anxiety-like behaviors. To verify this abnormality, we performed the elevated plus maze (EPM) tests (Figures 1D–1F, S1H, and S1I), another assay for evaluating anxiety behaviors, at the 4th week after rotenone exposure. Rotenone-treated mice showed a significant reduction in total travel distance (Figure S1H), consistent with the impaired locomotion in the open field results (Figure 1C). Importantly, these mice displayed a significantly lower number of open-arm entries and less time (Figures 1E and 1F) and travel distance in the open-arm (Figure S1I). These results suggest that rotenone induced non-motor deficit at this acute stage, a psychiatric symptom often observed in patients with PD before the onset of motor dysfunction.

### Rotenone exposure disrupts motor balance, coordination, and motor learning within 4 weeks

Next, we examined our PD model for the changes in motor balance and coordination. Male mice were trained and evaluated using the rotarod task right after the day of completing chronic rotenone exposure (Figures 1G–1I). The results showed that rotenone-treated mice performed significantly worse than controls, with a significantly shorter latency to fall, suggesting severely impaired motor balance and coordination during the task (Figure 1G). Further, while the control group improved its performance significantly 3 weeks later as compared to the initial level (baseline), the rotenone-treated group showed worse performance (Figures 1H and 1I). These distinct time-dependent changes between the two groups may stem from different motor learning, which was impaired in the rotenone-treated group. Additional Y-maze tests showed no difference between the two groups (Figure 1J), indicating intact spatial memory in the rotenone group. Together, these data suggest that rotenone treatment significantly impairs motor balance and coordination, as well as preferentially disrupts motor learning.

### Cellular changes in TH expression levels and TH^+^ and NeuN^+^ neuron density within 4 weeks

SNc is a histologically continuous structure composed of genetically and functionally diverse DA neurons in the midbrain,^27^^,^^28^^,^^29^^,^^30^ and patients with PD show prominent lesions in this region. We first examined DA lesions during the acute phase. Rotenone induced a strong reduction of tyrosine hydroxylase (TH) fluorescence in the whole SNc (Figures 2A and 2B), particularly in the middle ventral region of the SNc (Figure S1J). The latter change recapitulates the subregional selectivity of degeneration at the ventral SNc in human PD,^31^ which is partially associated with distinct gene expression of SOX6^+^/Aldha1^+^ (but lacks Otx2).^28^^,^^29^ TH^+^ cell number and density in the SNc decreased by ∼40% in rotenone-treated mice as compared with controls (Figures 2C and 2D). To confirm that TH^+^ neuronal loss was not limited to a single representative section, stereological analysis was performed using three serial coronal sections per mouse spanning the SNc. All analyzed sections consistently showed significantly reduced TH^+^ neuron density in rotenone-treated mice, confirming widespread DA degeneration (Figures S2A–S2C).

**Figure 2:**
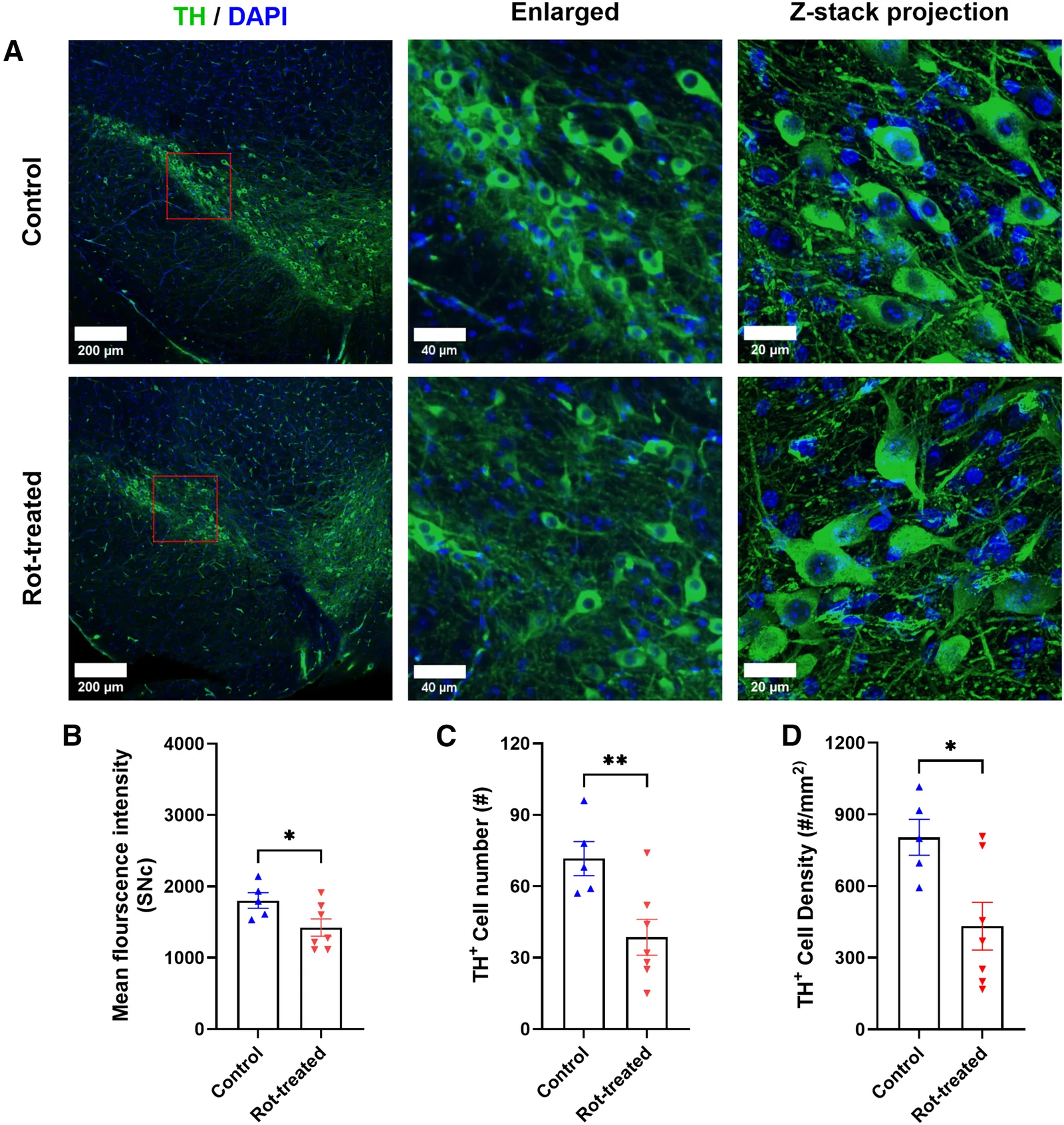
Rotenone exposure causes TH^+^ neuron loss in the SNc at the acute phase (A) Representative confocal images of tyrosine hydroxylase (TH, green) with nuclear counterstain (DAPI, blue) in control and rotenone-treated mice. (B–D) SNc TH fluorescence intensity (B), TH^+^ neuron numbers (C), and density (D). TH^+^ neurons were counted when co-stained with DAPI, and regions of interest (ROIs) were drawn manually based on TH staining and local anatomy in Fiji (see STAR Methods). Control *n* = 5, Rot-treated *n* = 7. Data are represented as mean ± SEM. ∗*p <* 0.05, ∗∗*p* < 0.01; two-tailed Welch’s *t* tests. Scale bars, 200 μm (left), 40 μm (middle), and 20 μm (right).

To further validate DA neuronal degeneration, we performed TH and NeuN co-immunostaining in the substantia nigra. While TH labels DA neurons, NeuN serves as a general neuron marker, and the TH^+^/NeuN^+^ ratio provides an estimate of the remaining DA neuron portion within the SNc region. Following 3 weeks of rotenone exposure, NeuN^+^ neuron density, however, was unchanged between control and Rot-treated mice (Figures 5A and 5B), indicating that total neuronal density was preserved. In contrast, the TH^+^/NeuN^+^ cell ratio was significantly reduced in rotenone-treated mice (Figure 5C), demonstrating selective loss of TH^+^ neurons possibly due to diminished TH expression––phenotypic impairment of DA neurons rather than global neuronal loss. Interestingly, unlike SNc, striatum TH fluorescence was not changed, even in the dorsal lateral striatum that receives prominent SNc axonal projection^32^ (Figures S3A–S3C). High-resolution imaging at this subregion confirmed the presence of intact TH^+^ axons and their varicosities. This is consistent with variable findings reported in striatal DA fibers^33^ and their compensatory sprouting in neonatal mice^34^ but contrasts with striatal DA axon loss and hypertrophy in rats^17^ with a delayed SNc lesion seen in the 6-OHDA model^35^^,^^36^ and MPTP model,^37^^,^^38^^,^^39^ implying a different toxicity in these PD models.

### Deficits in locomotion and anxiety behaviors are resolved by 12 months

After establishing robust Parkinsonism in the acute phase, we tracked their long-term changes at 12 months after toxin cessation. To our surprise, most of the deficits observed in the acute phase were resolved, and the rotenone-treated mice behaved normally as controls. As shown in the open field tests, rotenone-treated mice showed similar locomotion to controls (Figure 3). Both groups exhibited comparable outcomes in travel distance (Figures 3A and 3B), average speed, and mobility time (Figures S7A and S7E). Anxiety-like behavior, as measured by inner zone distance (Figure 3C) and outer zone distance (Figure S7B), of rotenone-treated mice was equal to that of controls. The longitudinal results within 12 months revealed a full picture of rotenone toxicity in the short and long terms (Figure 3D). While locomotion was strongly impaired in the acute phase, it fully recovered to the control levels within ∼4 months.

**Figure 3:**
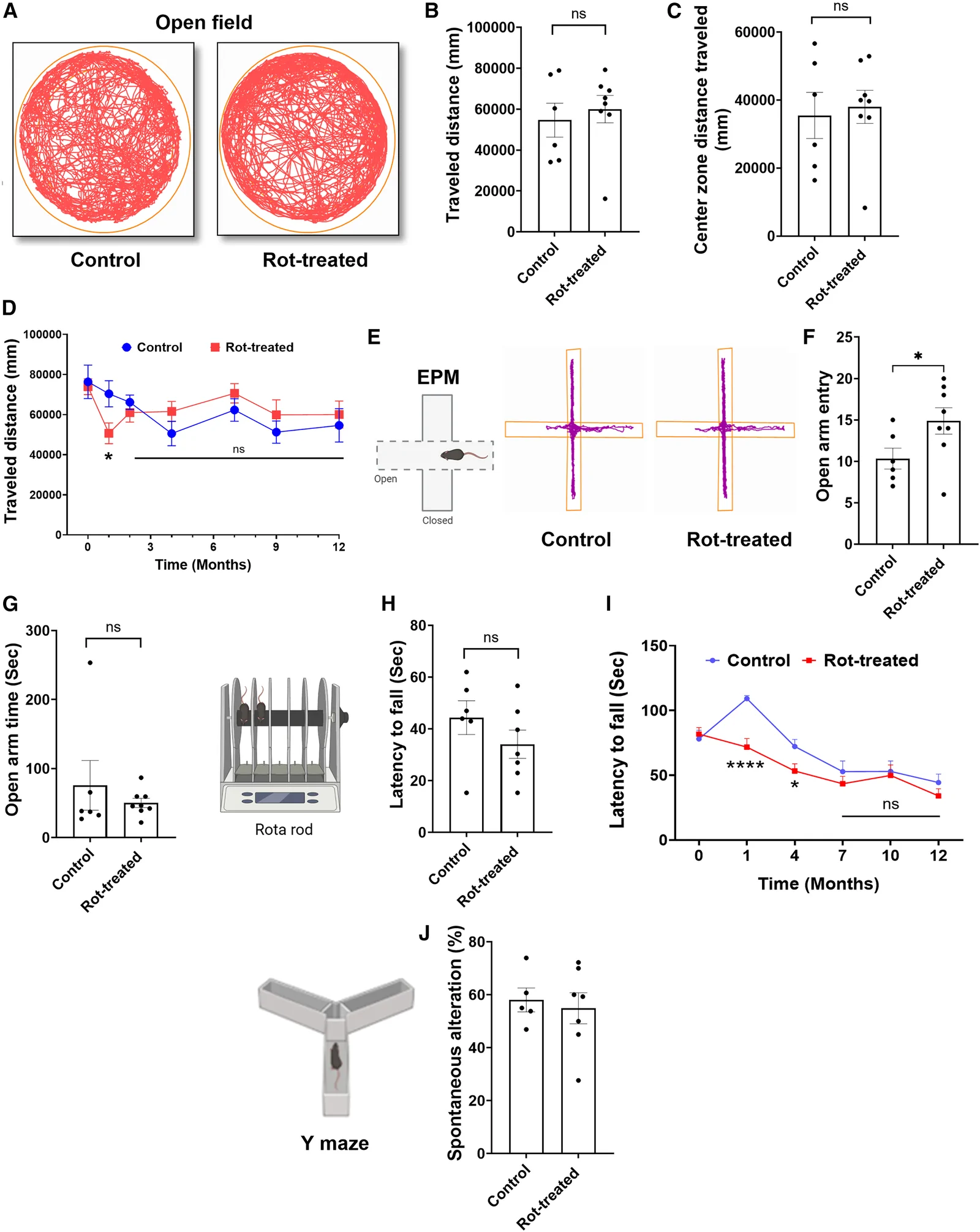
Resolution of rotenone-induced locomotor deficits, anxiety-like behaviors, and motor deficits at the long-term stage (A) Representative movement tracks in the open field test from a control and a Rot-treated mouse at 12 months following 3 weeks of rotenone exposure. (B and C) Quantification of total distance traveled (B) and center zone distance traveled (C). (D) Longitudinal analysis of the total distance traveled in open field tests. The first point represents the baseline before treatment (“0-month” point). (E) Representative mouse trajectories in the elevated plus maze (EPM). (F and G) Quantification of open-arm entries and time in the EPM. (H) Latency to fall in the rotarod test at 12 months. (I) Longitudinal analysis of rotarod task performance at different times. The significantly worse performance in the Rot-treated mice at 1 and 4 months, but similar performance at 7 months and later. (J) Spatial working memory assessed by the Y-maze test at 12 months. Data are presented as mean ± SEM, with individual points representing single animals. Statistical analyses were performed using two-tailed Welch’s *t* tests, except for the longitudinal analyses (D and I), which were analyzed using two-way repeated-measures ANOVA with Sidak’s multiple comparisons test. Sample sizes: open field, EPM, and 12-month rotarod: control *n* = 6, Rot-treated *n* = 8; Y-maze: control *n* = 5, Rot-treated *n* = 7; longitudinal analyses, baseline to 1 month: control *n* = 12, Rot-treated *n* = 16; 1–7 months: control *n* = 6, Rot-treated *n* = 10; 9–12 months: control *n* = 6, Rot-treated *n* = 8. Statistical significance is indicated as ∗*p* < 0.05, ∗∗∗∗*p* < 0.0001, and ns, not significant.

EPM tests at 12 months further showed no difference between the two groups. They showed similar results in total travel distance (Figure S7H), open arm entry, time (Figures 3F and 3G), and distance (Figure S7I). This is consistent with the results in the open field tests showing indistinguishable inner/outer distances (Figures 3C, S7B, and S7C). As expected, the Y-maze tests showed no changes between the two groups in the acute phase.

These results indicate that rotenone-induced Parkinsonism, including impaired locomotion and non-motor function in the acute phase, is fully reversible in the long-term as compared to the control group. These findings uncover the previously unappreciated resilience and adaptability of the DA system.

### The impaired motor balance and coordination recover by 12 months

Next, we measure the long-term changes in motor balance and coordination using rotarod assays. Rotenone-treated mice performed the task equally well as control mice at 12 months (Figures 3H and 3I), suggesting the recovery of motor balance and coordination. The longitudinal analysis of motor function revealed interesting dynamic changes in the same mice within 12 months (Figure 3I). Rotenone treatment significantly worsened the performance of the rotarod task in the acute phase (after the 3^rd^ week), but the rotenone-treated mice became indistinguishable from the age-matched controls at 7 months. Notably, both groups exhibited a significant decline in motor performance over the course of the experiments. The transient increase of the control group at the 1^st^ month before performance decline may arise from the initial motor learning, which was impaired in the rotenone-treated group. The curve divergence of two groups at the acute phase speaks for the effectiveness of rotenone treatment, and their convergence at the later stage supports the long-term changes of rotenone-induced damage relative to normal age-dependent decline.

### The PD-like cellular pathology recovers by 12 months

Given the notion that adult brain neurons rarely regenerate and neurodegeneration is irreversible, the observed long-term recovery to control levels is surprising. To better understand its mechanism, we examined midbrain cellular changes at 12 months. Figure 4 shows TH fluorescence levels and TH^+^ cell density in the SNc between the two groups. In comparison, rotenone-treated mice still had lower levels of TH fluorescence than the age-matched controls (Figures 4A and 4B). However, TH^+^ neuron number and density in the SNc become similar to the control group (Figures 4C and 4D). These findings may partially explain the resolution of motor disruption. Notably, TH^+^ cell density in control SNc reduced significantly (∼45%) at 12 months (Figure 4D) as compared with that at 1 month (Figure 2D). This is consistent with the age-dependent decline (>30%) of SNc TH^+^ neuron numbers reported in rats^40^ and humans.^31^^,^^41^^,^^42^ However, this age-dependent decline of TH^+^ cell density in the rotenone-treated group was nearly abolished (Figures 2D and 4D). Additionally, TH levels from the dorsal lateral striatum remained similar between the two groups (Figures S3D–S3F).

**Figure 4:**
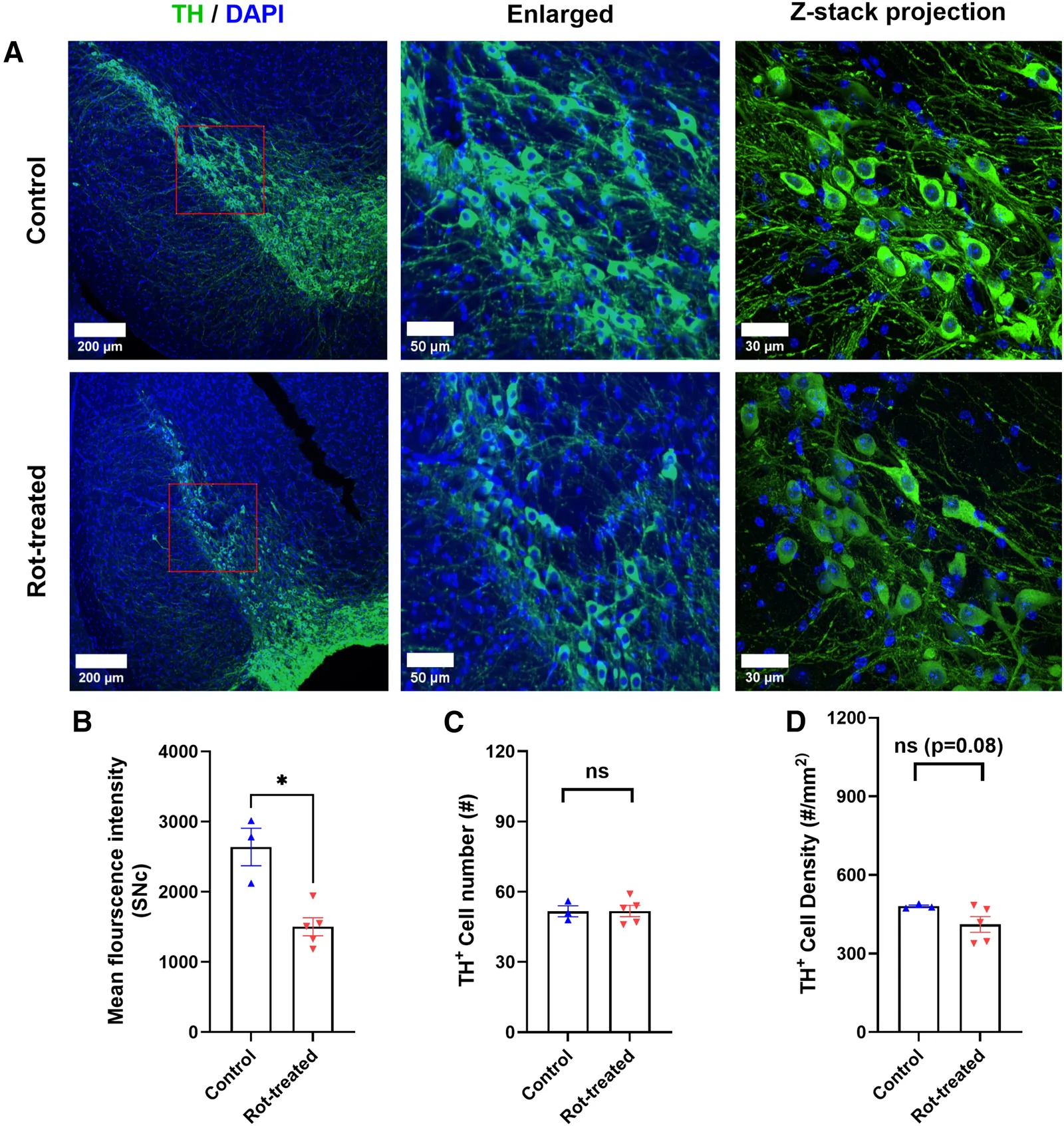
Long-term changes of dopaminergic neurons in the SNc at 12 months (A) Representative confocal images of midbrain sections stained with TH^+^ (green) and nuclei (DAPI, blue) from control and rotenone-treated groups at 12 months. (B–D) Quantification of TH expression levels (B), TH^+^ cell counts (C), and TH^+^ cell density (D) in the SNc region Mean ± SEM, two-tailed Welch’s *t test*; control *n* = 3, Rot-treated *n* = 5; *∗ p* < 0.05; ns, not significant. Scale bars, 200 μm (left), 50 μm (middle), and 30 μm (right).

To evaluate the long-term impact of rotenone on SNc neuron density, we performed TH/NeuN co-staining at 12 months (Figure 5D). The results showed that rotenone-treated mice had a similar number of NeuN^+^ cell density (Figure 5E), and the TH^+^/NeuN^+^ ratios in SNc were also indistinguishable (Figure 5F).

**Figure 5:**
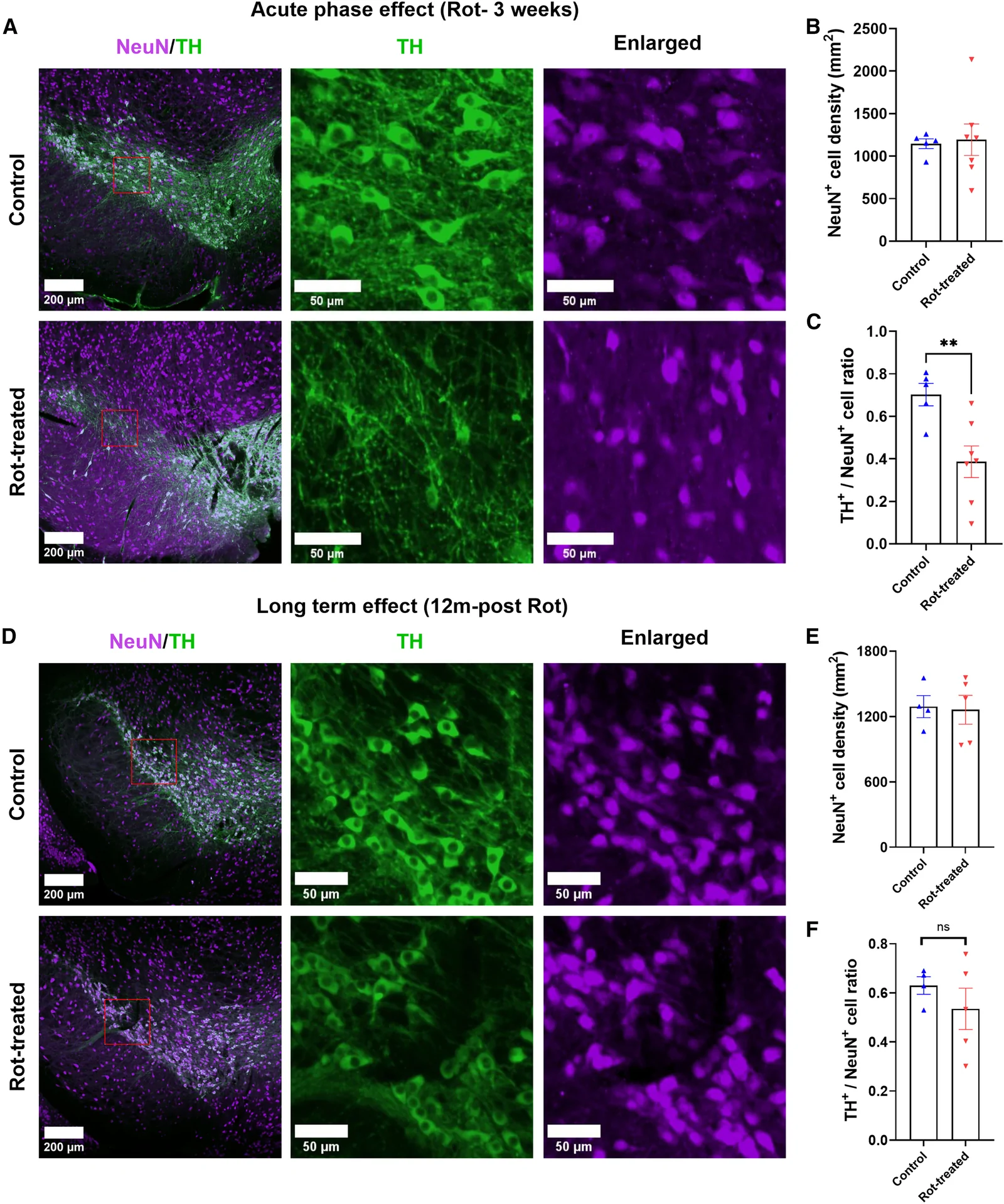
TH^+^/NeuN^+^ neuronal population changes at the acute phase and at 12 months (A) Representative confocal images of middle brain sections stained with NeuN^+^ (magenta) and TH^+^ (green) from control and Rot-treated mice following acute rotenone exposure. (B) Quantification of NeuN^+^ cell density in the SNc. (C) Quantification of the TH^+^/NeuN^+^ cell ratio in the SNc following acute rotenone exposure. (D–F) Like (A)–(C), but after long-term recovery. Mean ± SEM, two-tailed Welch’s *t* test; acute phase effect (Rot-3 weeks), control *n* = 5, Rot-treated *n* = 7; long-term effect (12 months post-Rot), control *n* = 4, Rot-treated *n* = 5;*∗∗p <* 0.01. Scale bars, 200 and 50 μm, as indicated.

Collectively, these data demonstrated that at 12 months after rotenone exposure, while SNc TH expression levels remained lower, TH^+^ cell density and TH^+^/NeuN^+^ ratio in the rotenone-treated group reached the levels of age-matched, untreated controls.

### Undetectable neuroinflammation at both acute phase and late stage

Microglial activation and astrocytic responses are key contributors to neuroinflammation in PD.^43^ We estimated the potential changes of neural inflammation using the expression of microglia protein Iba1 (ionized calcium-binding adapter molecule 1) and astrocyte GFAP (glial fibrillary acidic protein) in the SNc.^43^^,^^44^ At the acute phase, confocal imaging revealed normal microglia morphology and expression (Figure 6), which was further confirmed at high-resolution 3D images (not shown). We observed no microglial dystrophy and no other visible difference between control and Rot-treated mice (Figure 6A). Quantitative analysis showed no significant difference in Iba1 intensity or positive area (Figures 6B and 6C). Similarly, GFAP staining demonstrated no significant astrocytic activation in rotenone-treated mice compared to controls, as reflected by similar levels of GFAP intensity and area (Figures 6D–6F). Similar results were found in the dorsal-lateral striatum at the acute stage (Figures S4A–S4F). Next, we examined microglia changes using purinergic P2RY12 receptor (P2RY12), another microglia marker that is exclusively expressed on microglia and required for microglia migration and response, with its levels reduced and even lost upon microglia dystrophy.^45^^,^^46^ We found no apparent changes in P2RY12 fluorescence intensity and microglia density (Figure S5), implying the absence of neuroinflammation in this model. This is unexpected since other PD models induced by lipopolysaccharide (LPS)^47^ or α-synuclein fibril injection^48^ showed microglia activation. These data suggest the absence of neural inflammation in the current rotenone mouse model.

**Figure 6:**
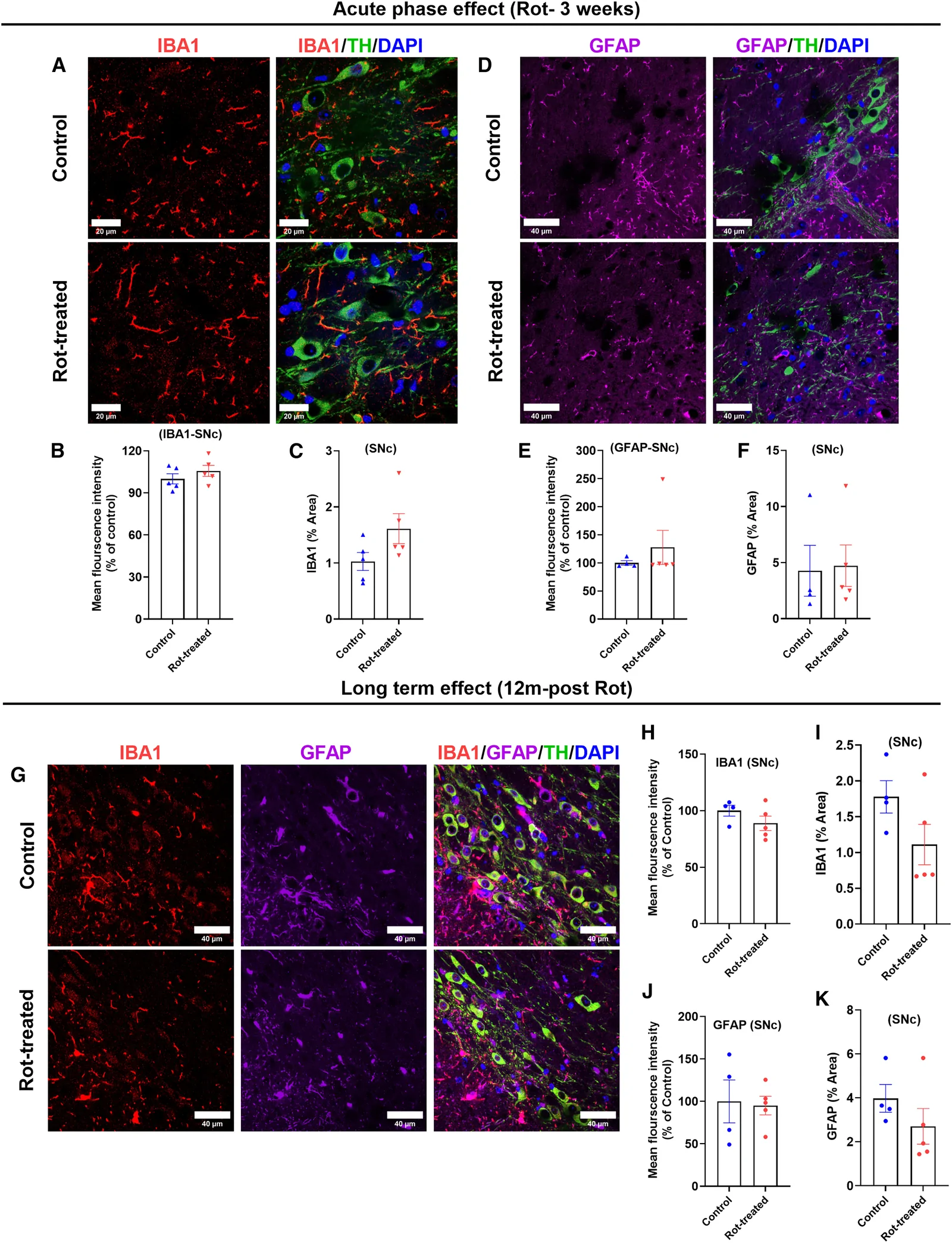
Rotenone exposure does not significantly alter neuroinflammation in the SNc (A and D) Representative confocal images of the substantia nigra stained for Iba1 (red), tyrosine hydroxylase (green), GFAP (magenta), and DAPI (blue) from control and Rot-treated mice during the acute phase (A and D). (B and C) Quantification of Iba1-MFI and Iba1-positive areas in the SNc during the acute phase. (E and F) Quantification of GFAP MFI and GFAP-positive area in the SNc during the acute phase. (G) Confocal images at the late stage (12 months). (H and I) Quantification of IBA1 MFI and area in the SNc at the long-term stage. (J and K) Quantification of GFAP MFI and area in the SNc at the late stage. Data are presented as mean ± SEM. Statistical analysis was performed using an unpaired two-tailed Welch’s *t* test. Sample sizes: acute phase effect (Rot-3 weeks) (control *n* = 5, Rot-treated *n* = 5); long-term effect (12 months post-Rot) (control *n* = 4, Rot-treated *n* = 5). Scale bars, 20 and 40 μm, as indicated.

At 12 months, we examine whether there is delayed neuroinflammation. Confocal imaging in the SNc region showed no difference in SNc microglia morphology and Iba1 expression between the two groups (Figures 6H and 6I). GFAP signal also showed no significant astrocytic activation in rotenone-treated mice compared to controls (Figures 6J and 6K). Similar results were found in the striatum (Figures S4G–S4K). To cross-validate these findings, we examined P2RY12, another microglia hemostatic marker. While the SNc showed slightly higher P2RY12 levels than the substantia nigra pars reticulata (SNr), the SNc signals were comparable between the two groups of mice (Figures S8A–S8E). Interestingly, SNr P2RY12 cell number and density were both lower in rotenone-treated mice than in controls (Figures S8F and S8G), suggesting partial microglia activation in the SNr region. Dorsal striatal imaging revealed similar P2RY12 levels and microglia density between the two groups (Figures S8H–S8J). These data showed consistent results in this mouse model, demonstrating the absence of neuroinflammation in both the acute phase and the late stage of rotenone exposure.

### Phosphorylation of α-synuclein (Ser129) at the acute phase

Phosphorylation of α-synuclein at Ser129 (pS129-α-Syn) is a critical post-translational modification^49^^,^^50^ found in most α-synuclein deposits and heavily linked to synucleinopathies in PD. We next examined whether pS129-α-Syn accumulates in the SNc and striatum regions using immunostaining assays. At the acute phase, we observed basal immunoreaction of the antibody in both control and treated mice, but there was no significant difference between the two groups, as quantified in pS129-α-Syn fluorescence intensity and pS129-α-Syn-positive area (%) in both the SNC and striatum (Figures S6A–S6F). Similarly, the same results were found at 12 months in both the SNc and striatum regions (Figures S6G–S6L).

At 12 months, there was an increasing trend of pS129-α-Syn immunoreactivity. 3 out of 5 Rot-treated mice showed higher pS129-α-Syn mean fluorescence intensity and positive area in both the SNc and striatum relative to controls. Nevertheless, intergroup comparisons did not reach statistical significance for either parameter in the SNc or striatum (Figures S6G–S6L). Future studies with larger sample numbers are required to further explore the involvement of pS129-α-Syn.

## Discussion

This work examined the long-term effect of systemic rotenone exposure in mice and the time-dependent changes of Parkinsonism after rotenone cessation. We first established robust PD phenotypes in the acute toxicity phase and then monitored their longitudinal changes for 12 months. The results uncover a surprising reversibility and resilience of the impaired motor and non-motor function, which has direct implications for understanding the long-term compensation and plasticity of the DA system in PD. To our knowledge, this is the first study to examine the longitudinal effects of rotenone in mice.

Rotenone models have been increasingly used to mimic progressive features of PD^9^ since their first demonstration in Sprague-Dawley and Lewis rats.^8^ While rat rotenone models have been well studied,^9^^,^^17^^,^^51^ mouse rotenone models have been less explored, with mixed results reported.^11^ The oral rotenone mouse model was used to study PD^16^^,^^23^^,^^52^ and to evaluate therapeutics,^53^^,^^54^^,^^55^ but its effectiveness as a PD model was challenged recently.^26^ Other routes of exposure, such as subcutaneous administration at low doses (2.5–4 mg/kg/day),^56^ i.p. (1–10 mg/kg/day for 3 weeks),^57^ or environmental exposure via breeding cages (5 mg/kg/day for 2–6 weeks),^22^ have also been explored for modeling PD. Consistent with these works, here we demonstrated that rotenone i.p. injection induced robust Parkinsonism in the acute exposure phase. We showed that chronic rotenone i.p. injections gradually induced PD-like deficits, including hypokinesia, impaired motor coordination and motor learning, as well as non-motor dysfunction such as increased anxiety. Along with these functional deficits, the cellular pathology becomes prominent in parallel, including TH^+^ neuron loss in the SNc and reduced TH expression. Interestingly, these changes in TH were confined to the SNc, without changes in the striatum. This may be partially attributed to multiple origins of TH^+^ axons at the striatum or compensatory axon fiber sprouting.^34^ Notably, our i.p. model displayed a much higher animal survival rate than prior studies, with nearly all the mice surviving except for a few that were euthanized for non-specific conditions. This contrasts with the 85% mortality within 56 days of oral rotenone exposure previously reported,^16^ implying lower non-specific toxicity of rotenone in this PD model.

Most rodent studies focused on the rotenone effect in the acute phase, leaving its long-term consequences unclear. Here, we performed a longitudinal study for 12 months after rotenone exposure for 3 weeks. We found that the Parkinsonism developed in the acute phases, such as locomotion deficit, anxiety, and motor balance and coordination, was fully reversible in the long term, and the treated mice could perform motor tasks as well as age-matched controls. These results provide the first direct evidence on the adaptation and resilience in PD-associated neural circuits. The underlying mechanisms remain unclear. It may involve compensatory neural plasticity or circuitry remodeling linked with motor and non-motor deficits and the stabilization/resilience of treated DA neurons; further exploiting these mechanisms may gain new insights into how to slow PD progression. This is the first study in mice that tracks PD onset, development, and reversal for over a year. Additionally, it reveals an age-dependent decline in motor performance in both control and Rot-treated group. The initial improvement of motor performance in the control group but not in the rotenone group may be attributed to differences in motor learning. While the performance curve divergence between the two groups speaks for the effect of rotenone treatment at the acute phase, the curve convergence at the later stage illustrates the long-term motor function changes. The latter result reflects a previously unknown stabilization of SNc DA neurons in rotenone-treated mice that contrasts to a significant age-dependent decline (∼45%) in the control group.

The comparable TH^+^ neuron density between control and treated mice at the long term is surprising since neurodegeneration was thought to be irreversible, and most adult brain neurons cannot regenerate.^58^^,^^59^ On the other hand, it becomes increasingly clear that the dentate gyrus and subventricular zone retain the ability of adult neurogenesis under physiological conditions.^58^^,^^60^ Further, certain pathological conditions (e.g., brain ischemic injury) can activate neurogenesis in the hippocampus.^59^^,^^61^^,^^62^^,^^63^ Recent work reported that both adult serotonin^64^ and norepinephrine neurons^65^ possess the intrinsic ability to regrow their axons upon injury.^66^ DA axons share some similarities in the long-range, extensive branching and spreading projection with modulatory roles, but it is unclear whether their axons in the striatum may regain a similar ability for axon regrowth or compensatory re-sprouting upon rotenone-induced stress. Indeed, neonatal SNc lesions induced by 6-OHDA unilateral injection have substantial capacity for compensatory sprouting of DA axons,^34^ leading to less TH reduction in the striatum than in the SNc. The SNc region contains heterogeneous DA neurons,^27^^,^^29^^,^^30^ with highly diverse genetic profiles. It is unclear if chronic rotenone exposure may reprogram certain DA neurons to regain regeneration ability or alter their cellular identity. Our data showed a clear TH^+^ neuron loss and intact NeuN^+^ density in the SNc in the acute phase of rotenone exposure, suggesting an aberrant reduction or abolishment of TH expression that may arise from stress-induced metabolic reprogramming in those DA neurons rather than neuron death. Further addressing the underlying mechanism is important to understanding the resilience of DA neurons and developing new interventions in PD conditions.

Neuroinflammation has been involved in PD pathology. In the rotenone model of PD, inflammation in the SNc and striatum, as reflected by changes in Iba1 and GFAP, demonstrates variability depending on the model used. In rat models, rotenone administration significantly increased Iba1 and GFAP expression in the SNc.^18^^,^^25^^,^^67^ In mouse models, rotenone treatment increased Iba1 and GFAP immunoreactivity in the SNc^21^^,^^68^ with a regional difference at striatum. Our data found no significant difference in Iba1, GFAP, and P2RY12 signaling in the SNc or striatum throughout the acute and late phase, suggesting the absence of neuroinflammation in this model. It appears that neuroinflammation in the PD models is context dependent, depending on species, route of administration, rotenone dosage, and subregion analyzed.

The accumulation of pS129-α-Syn in the SNc and striatum has been documented in rotenone PD models,^20^^,^^69^^,^^70^ which is model and context dependent.^71^ We found no significant changes in pS129-α-Syn in either the SNc or the striatum in our model. This is consistent with findings reported in the previous study^72^ but different from the rat models.^17^

The progressive development of motor deficit and loss of TH^+^ neurons during the acute phase supports a critical role of mitochondrial dysfunction in PD pathogenesis, which is aligned with the positive correlation between pesticide exposure history and PD risk. However, the recovery of these deficits after rotenone withdrawal uncovers a limited long-term consequence of rotenone exposure. While this is consistent with the reversibility of rotenone binding to mitochondrial complex I, it challenges the long-held notion of pesticide exposure history as a cause of PD. Indeed, while epidemiological studies support a generic correlation between pesticide exposure history and PD incidence, numerous methodological limitations prevent a definite conclusion on the causal role of the exposure.^5^^,^^6^ In our model, the chronic, systemic, and high-dose rotenone exposure only induces transient Parkinsonism rather than permanent deficits in mice, suggesting strong resilience and adaptability of the DA system. Together, this work has established the effectiveness of rotenone exposure in inducing Parkinsonism and further evaluated the long-term effects of rotenone exposure. The longitudinal data reveal the reversibility of PD-like deficits in the long term, uncovering a previously underappreciated capacity of resilience and adaptation of the DA system *in vivo*, which has the potential to be exploited for preventing PD progression.

### Limitations of the study

First, this work is about a toxin-based longitudinal model designed to study the long-term resilience and adaptive capacity of the nigrostriatal system rather than a genetic recapitulation of progressive human PD. Despite being useful to study mitochondria-centric pathology of PD, the rotenone-induced mouse PD model used here does not fully recapitulate human PD phenotypes, and it does not display typical α-synuclein-centered pathology and lysosomal-related mechanism as in human PD. Further, as a reversible blocker of mitochondrial complex I, systemic rotenone exposure may also induce muscle weakness and malaise in addition to the brain dysfunction, which may contribute to observed behavior assays. Variability has been noted in literature, which could arise from rotenone handling (as it is prone to degrade when exposed to light and water), dosages, sex-dependent metabolism, and pharmacokinetics (which is faster in male rodents).^11^ Second, the species-specific differences between mice and humans need to be kept in mind. Mice have much shorter lifespans and smaller cell sizes, and they also exhibit critical molecular differences in the PD context, such as different dopamine metabolism,^73^ lack of neuromelanin, divergent properties in α-synuclein and LRRK2,^74^ and glia gene profiles.^75^^,^^76^^,^^77^ Thus, future work is needed to verify in what extent these results can be extrapolated to humans.

## Resource availability

### Lead contact

Further information and requests should be directed to and will be fulfilled by the lead contact, Dr. Xuelin Lou (xulou@mcw.edu).

### Materials availability

This study did not generate new, unique reagents.

### Data and code availability

• The raw data used to generate the figures and tables will be made available from the lead contact upon reasonable request.
• This paper does not report original code.
• Any additional information required to reanalyze the data reported in this paper is available from the lead contact upon reasonable request.

## Acknowledgments

We are thankful to Rodent Behavior Core at the Neuroscience Research Center at the Medical College of Wisconsin for assistance with behavioral testing. The funding for the purchase of core equipment was provided by the Research and Education Initiative Fund, a component of the Advancing a Healthier Wisconsin Endowment at the Medical College of Wisconsin. We thank the Oxford Instruments Center for Advanced Microscopy and the EM core for imaging experiments and analysis. The Facility Line STED microscope was purchased via an NIH high-end Instrument S10 Award (S10OD034247-01A1 to X.L.). We thank other members Dr. Sandeep Kumar Kotturu, Michael Abiola, Adeel Ahmad, and Claire Wulf of the Lou Lab for technical support and comments on the manuscript.

This work is supported partially by awards from the 10.13039/100000002National Institutes of Health (NIH, R01DK132088, R01DK133326, R01AG079257, and S10OD034247-01A1 to X.L.), the 10.13039/100008980Medical College of Wisconsin (to X.L.), and 10.13039/100021528Advancing a Healthier Wisconsin Endowment (FP00028084 to P.J.). P.J. is partially supported by the AHW Post-doc Research Seed Grant.

## Author contributions

P.J. designed the study, performed the experiments, analyzed the data, and wrote the manuscript; F.F. designed the study and performed the experiments; X.L. directed the study, designed the experiments, analyzed and interpreted the data, and wrote the manuscript.

## Declaration of interests

The authors declare no competing interests.

## STAR★Methods

### Key resources table

**Table:** 

REAGENTS or RESOURCE	SOURCE	IDENTIFIER
**Antibodies**		

Anti-TH antibody	Millipore	Cat# AB152; RRID:AB_390204
Anti-TH antibody	Immunostar	Cat# 22941; RRID:AB_572268
Anti-TH antibody	SYSY	Cat# 213 104; RRID:AB_2619897
Anti-P2RY12 antibody	BioLegend	Cat# 848002; RRID:AB_2650634
Anti-pS129-α-Syn antibody	Abcam	Cat# ab51253; RRID:AB_869973
Anti-NeuN antibody	Millipore	Cat# ABN78; RRID:AB_10807945
Anti-Iba1 antibody	Wako-chem	Cat# 015-28011; RRID:AB_3094971
Anti-GFAP antibody	NeuroMab	Cat# MA5-45632; RRID:AB_2932086
Donkey anti-Mouse IgG (H+L) Highly Cross-Adsorbed Secondary Antibody, Alexa Fluor™ Plus 488	Invitrogen	Cat# A32766; RRID:AB_2762823
F(ab')2-Goat anti-Mouse IgG (H+L) Cross-Adsorbed Secondary Antibody, Alexa Fluor™ 647	Invitrogen	Cat# A-21237; RRID:AB_2535806
Donkey anti-Rabbit IgG (H+L) Highly Cross-Adsorbed Secondary Antibody, Alexa Fluor™ Plus 488	Invitrogen	Cat# A32790; RRID:AB_2762833
Goat anti-Rabbit IgG (H+L) Highly Cross-Adsorbed Secondary Antibody, Alexa Fluor™ 568	Invitrogen	Cat# A-11036; RRID:AB_10563566
Goat anti-Rabbit IgG (H+L) Highly Cross-Adsorbed Secondary Antibody, Alexa Fluor™ 647	Invitrogen	Cat# A-21245; RRID:AB_2535813
Goat anti-Guinea Pig IgG (H+L) Highly Cross-Adsorbed Secondary Antibody, Alexa Fluor™ 488	Invitrogen	Cat# A-11073; RRID:AB_2534117
Goat anti-Guinea Pig IgG (H+L) Highly Cross-Adsorbed Secondary Antibody, Alexa Fluor™ 568	Invitrogen	Cat# A-11075; RRID:AB_2534119
Goat anti-Guinea Pig IgG (H+L) Highly Cross-Adsorbed Secondary Antibody, Alexa Fluor™ 647	Invitrogen	Cat# A-21450; RRID:AB_2535867
Donkey anti-Rat IgG (H+L) Highly Cross-Adsorbed Secondary Antibody, Alexa Fluor™ 488	Invitrogen	Cat# A-21208; RRID:AB_2535794
Donkey anti-Rat IgG (H+L) Highly Cross-Adsorbed Secondary Antibody, Alexa Fluor™ 594	Invitrogen	Cat# A-21209; RRID:AB_2535795
Donkey Anti-Mouse IgG (H+L), Highly Cross-Adsorbed (Min X Rat) (CF 568)	Biotium	Cat# 20105-1; RRID:AB_10853136
Donkey Anti-Mouse IgG (H+L), Highly Cross-Adsorbed (Min X Rat) (CF 640)	Biotium	Cat# 20177; RRID:AB_10853475
Donkey Anti-Rabbit IgG (H+L), Highly Cross-Adsorbed (Min X Rat) (CF 568)	Biotium	Cat# 20098; RRID:AB_10557118
Donkey Anti-Rabbit IgG (H+L), Highly Cross-Adsorbed (Min X Rat) (CF 640)	Biotium	Cat# 20178-1; RRID:AB_10852688

**Chemicals, peptides, and recombinant proteins**		

Rotenone	Sigma-Aldrich	Cat# R8875
DMSO	Sigma-Aldrich	Cat# 276855
Miglyol 812	Medisca	Code# 207878B
DAPI	Thermo Fisher	Cat# 62248
ProLong Gold	Thermo Fisher	Cat# P36930

**Experimental models: Organisms/strains**		

C57BL/6J mice	The Jackson Laboratory	Strain# 000664

**Software and algorithms**		

Fiji (ImageJ)	ImageJ – Fiji	https://imagej.net/software/fiji/downloads
ANY-maze software	Stoelting	Version 7.8
GraphPad Prism	GraphPad Software	Version 10.6.0

**Other**		

Rotarod treadmill	IITC Life Sciences	Product #755
Elevated Plus Maze	San Diego Instruments	Product #7001-0316
Y-maze	San Diego Instruments	Part #7001-0424Y

### Experimental model and study participant details

#### Animals and housing

All experiments were performed on age-matched male C57BL/6J mice (Jackson Laboratory, USA). Mice were housed in a pathogen-free, temperature-controlled (22 ± 2°C) and humidity-controlled (45–60%) facility under a regular 12 h light/dark cycle, with standard polycarbonate cages in the cages. All cages were supplied with paper bedding and nesting materials. Food (standard rodent chow; LabDiet 5001) and water were available *ad libitum*. Mice showing severe weight loss (>20% of baseline) or distress were excluded by institutional animal welfare protocols. All procedures were approved by the Institutional Animal Care and Use Committee (IACUC) of the Medical College of Wisconsin and followed the NIH Guide for the Care and Use of Laboratory Animals (AUA00006329).

Male mice at 2 months of age were used for experiments since males produced consistent results and were less variable than females.^11^^,^^24^^,^^25^ This is consistent with our initial pilot experiments in female mice, showing that they were insensitive to rotenone exposure (data not shown). This is unexpected given faster rotenone metabolism in males than females.^11^ Mice were acclimated to the behavioral core facility for at least 2 weeks to minimize environmental impact. To further reduce non-specific behavioral perturbation, cohorts were randomized into two groups with intraperitoneal (IP) rotenone as the PD model and vehicle as controls, housed together throughout the study, and tested side-by-side in parallel and by the same well-trained experimenter for all animals.

### Method details

#### Rotenone preparation and dosing

Rotenone (Cat#R8875, Sigma-Aldrich, USA) was administered to C57BL/6 mice via intraperitoneal injection. Briefly, rotenone powder was dissolved in 100% dimethyl sulfoxide (DMSO) to generate a 50× stock solution, aliquoted into amber septa vials to avoid light and water exposure, and stored at −20°C. The stock solution was diluted in the medium-chain triglyceride Miglyol 812 (Code# 207878B, Medisca) to produce a final working solution of 2.5 mg/kg body weigh^17^ at a dosing volume of 10 μL/g body weight (98% Miglyol + 2% DMSO). The mixture was prepared fresh every other day. IP injections were administered once daily for 21 consecutive days. The dosage and frequency of rotenone is based on previous study rat study conducted at 2.0 mg/kg with a systemic complex I inhibition assay,^17^ confirming that rotenone once per 24 hours was appropriate (maximum complex I inhibition reached at 8 hours and recovery by 32 hours). Control mice received equivalent volumes of vehicle. Throughout treatment, mice were monitored daily for body weight, grooming, posture, mobility, and general health, with any adverse signs recorded and animals supported by institutional welfare guidelines.

#### Evaluation of motor and non-motor function

Open Field Test: Spontaneous locomotor activity was assessed in the open field tests (OFT). We used an apparatus consisting of four large circular arenas (diameter: 19 in, height: 12.5 in; corrugated baseboards). Before testing, animals were habituated to the behavioral suite for 30 minutes. Mice were individually placed in the center of the arena and allowed to explore freely for 30 minutes. Between each trial, arenas were thoroughly cleaned with 70% ethanol to remove olfactory cues. All experiments were conducted under standard lighting conditions with continuous white noise to minimize external disturbances. Mouse behavior was recorded by an overhead camera, analyzed using ANY-maze software (Stoelting Co., USA) for quantification of total distance traveled, average speed, time spent in the inner zones and outer zones, mobility, immobility, and freezing duration. Mice were assessed right before treatment (as the baseline) and then tested weekly to monitor deficit onset and progression, with the results measured shortly after the 3-week treatment as the acute phase. After that, mice were either used for cellular pathology characterization or kept for evaluating longitudinal recovery from 2 to 12 months and for pathological examination at the end of the study.

Elevated Plus Maze (EPM) Test. Anxiety-like behavior was assessed using the elevated plus maze (EPM; San Diego Instruments, USA; Product #7001-0316). The apparatus consists of two open arms (length: 12 in; width: 2 in; wall height: 15.25 in) and two closed arms (length: 26 in; width: 2 in; wall height: 21.25 in) arranged at right angles around a central platform. Mice were habituated to the behavioral suite for 30 minutes before testing, and tests were performed under standard lighting with continuous white noise. Each mouse was placed in the central platform facing an open arm and allowed to freely explore for 5 minutes. The maze was thoroughly cleaned with 70% ethanol between trials to eliminate olfactory cues. Behavior was recorded using an overhead camera and analyzed by ANY-maze software (Stoelting Co., USA) for automated scoring. Results were quantified using the total distance traveled in the maze, the number of open arm entries, time spent in the open arm, and the distance traveled in the open arm.

Rotarod Test. Motor coordination and balance were assessed using a Rotarod treadmill (IITC Life Sciences, USA; Product #755) equipped with rat drums (diameter: 3.75 in; lane width: 4 in; lane height from drum to base: 12 in). Before testing, animals were habituated to the behavioral suite for 30 minutes. Mice were placed individually on the rotating drum, and latency to fall was automatically recorded by the system. Testing was performed under an accelerating paradigm, with a start rotation speed of 4 rpm and continuously increased to 40 rpm over a 5-min trial. Each mouse underwent three trials per day, separated by 30-min inter-trial intervals, for three consecutive days. The third trial was used to analyze the final performance. Between each trial, the instrument was thoroughly cleaned with 70% ethanol to remove olfactory cues. All experiments were conducted under standard lighting conditions with continuous white noise to minimize external disturbances. Mice were tested right before treatment (as the baseline) and then tested weekly to monitor the deficit onset and progression, with the results measured shortly after the 3-week treatment as the acute phase. Mice were tested further after completion of rotenone exposure (IP, 2.5 mg/kg/day × 21 days) longitudinally from 2 to 12-month post-treatment. Latency to fall was averaged across trials for each mouse.

Y-Maze Test. Working memory was evaluated using the spontaneous alternation Y-maze paradigm (San Diego Instruments, USA; Part #7001-0424 Y). The maze was constructed of 0.25″ acrylic with clear walls and beige floors, consisting of three arms (length: 15 in; width: 3 in; wall height: 5 in) arranged at 120° angles. Mice were habituated to the behavioral suite for 30 minutes prior to testing, which was performed under standard lighting with continuous white noise to minimize external disturbances. Each mouse was placed individually at the end of one arm and allowed to freely explore all three arms for 10 minutes. Arm entries were recorded using an overhead camera system, and spontaneous alternation behavior was calculated as the number of sequential visits (ABC, ACB, BAC, BCA, CAB, CBA) to three different arms divided by the total possible alternations, expressed as a percentage. Between trials, the maze was thoroughly cleaned with 70% ethanol to remove olfactory cues.

#### Brain tissue preparation, immunofluorescence imaging and analysis

Brain tissues were prepared as previously reported,^44^^,^^78^ with minor modifications. Briefly, mice were euthanized by rapid decapitation in the middle of the 4^th^ week of rotenone exposure, and brains were immediately isolated and immersed in ice-cold phosphate-buffered saline (PBS). Whole brains were immersed in freshly prepared 4% paraformaldehyde (PFA) and 4% sucrose in PBS for 24 h at 4°C with gentle shaking. Following fixation, brains were transferred to 30% sucrose in PBS and stored at 4°C until sectioning. Coronal sections (30 μm) were cut using a vibratome, with the region of interest identified visually under a microscope and mounted on glass slides. The tissue sections encompassing substantia nigra pars compacta (SNc), substantia nigra pars reticulata (SNr), and striatum (ST) were identified by their cellular architecture and adjacent brain structures in the middle brain. SNc is an irregular-shaped nucleus concentrated with dopaminergic neurons, spanning a narrow, elongated region up to ∼1.4 x1.4 x 1 mm at medial-lateral, rostral-caudal, and dorsal-ventral directions (Allen Mouse Brain Atlas). In the coronal section of the middle brain, SNc typically displays as a “wing”-like structure, with dorsal SNc gradually merged with lateral Parabrachial pigmented nucleus (PBP), a part of the ventral tegmental area (VTA) toward the midline. Sections containing only part of SNc regions were not used for quantification of TH neuron loss. To minimize the subregional effect of slices, tissue sections from similar central SNc anatomic levels were used for comparison across individual mice and groups. Stereological study (every third slices, 30 μm/slice) confirmed that our method produces a reliable quantification of TH^+^ cell number and density in SNc (Figure S2).

For immunofluorescence staining, brain sections were washed in PBS and blocked for 1 h at room temperature in PBS containing 0.4% Triton X-100 and 5% normal goat serum. Sections were incubated overnight at 4°C with primary antibodies diluted in blocking buffer. The next day, sections were washed and incubated for 2 h at room temperature with secondary antibodies conjugated with Alexa Fluor dyes or CF-dyes (1:300, Invitrogen). Nuclei were counterstained with DAPI (1 μg/mL), and sections were mounted with ProLong Gold antifade mounting medium (Thermo Fisher). Primary antibody used here: rabbit anti-tyrosine hydroxylase (TH; Rabbit, 1:400, Millipore Cat#AB152, TH; mouse, 1:400, Immunostar Cat# 22941, TH; Guinea pig, 1:200, SYSY Cat#213104), P2RY12 (Cat#848002, 1:50, Biolegend), (pS129-α-Syn; 1:50, Abcam Cat#AB51253), (NeuN; Rabbit, 1:100, Millipore Cat#ABN78), (Iba1; Wako-chem, rabbit, 1:100), (GFAP; NeuroMab, mouse, clone N206A/8, 1:100).

Confocal imaging was performed primarily using an Andor BC-43 spinning disk confocal microscope (Oxford Instruments, UK) equipped with an sCMOS camera and objectives of 10×, 20×, and 60× oil. Some experiments were also imaged with a Nikon spinning disk confocal microscope, as described previously,^79^^,^^80^^,^^81^ and an Abberior STED microscope. For each batch of experiments, all images were acquired using identical acquisition parameters and settings (laser intensity, exposure time, and camera binning) to ensure consistency across samples. Images for comparison were presented with the same display settings.

Quantitative analysis was performed on 2D images, and regions of interest (ROIs) in the SNc, SNr, and striatum were delineated using the free-hand selection tool in ImageJ and Fiji. The ROIs of SNc were defined based on TH staining pattern and local neural anatomy, excluding the PBP and VTA complex. Striatum images were acquired from the comparable sites/areas at the dorsal lateral striatum from each coronal section, since this region is a major site of axonal projection from SNc dopaminergic neurons. Mean fluorescence intensity, percentage area of positive signal (% area), and cell density were measured within the ROIs of each dataset, and the results were averaged from different batches of experiments. TH^+^ neurons were visually identified by typical TH fluorescence spots diffusing around a nucleus.

### Quantification and statistical analysis

All data were presented as mean ± standard error of the mean (SEM), with individual data points representing biological replicates or individual mice. Statistical analyses were performed using GraphPad Prism (version 10.6.0; GraphPad Software, USA). Comparisons between two groups were conducted using a two-tailed Student’s *t*-test with Welch’s correction (Welch’s *t*-test) to account for unequal variances. For multi-group comparisons (e.g., column graphs), one-way or two-way analysis of variance (ANOVA) was applied, followed by Tukey’s multiple-comparisons post hoc test. For longitudinal or repeated time-point analyses (e.g., time-plot graphs), two-way ANOVA with Sidak’s multiple-comparisons post hoc test was used. Statistical significance was defined as ∗*p* < 0.05, ∗∗*p* < 0.01, ∗∗∗*p* < 0.001, ∗∗∗∗*p* < 0.0001.
