# Supplementary material for: A longitudinal study of rotenone-induced Parkinsonism in mice
Source: iScience. 2026 Oct 23;29(11):117996. doi: 10.1016/j.isci.2026.117996 (PMC13720679; doi:10.1016/j.isci.2026.117996)
Supplement: Supplementary file 1 [file mmc1.pdf]

iScience, Volume *29*

Supplemental information

A longitudinal study of rotenone-induced Parkinsonism in mice

Prince Joshi, Fan Fan, and Xuelin Lou

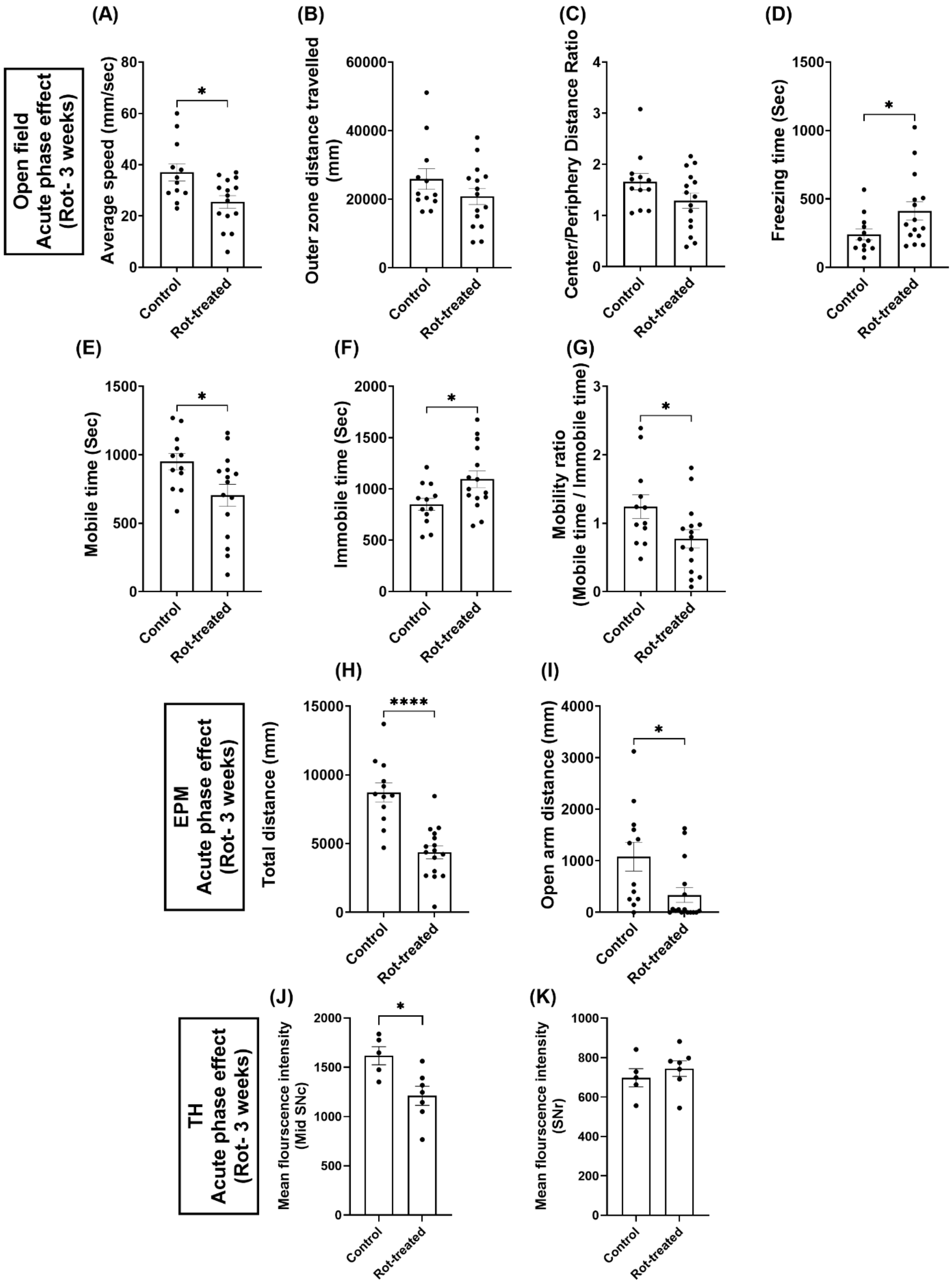
(A)
(B)
(C)
(D)
Open field
Acute phase effect
(Rot- 3 weeks)
Average speed (mm/sec)
Outer zone distance travelled (mm)
Center/Periphery Distance Ratio
Freezing time (Sec)
(E)
(F)
(G)
Mobile time (Sec)
Immobile time (Sec)
Mobility ratio (Mobile time / Immobile time)
(H)
(I)
EPM
Acute phase effect
(Rot- 3 weeks)
Total distance (mm)
Open arm distance (mm)
(J)
(K)
TH
Acute phase effect
(Rot- 3 weeks)
Mean flourscence intensity (Mid SNc)
Mean flourscence intensity (SNr)
Control
Rot-treated
*
****

Figure S1. Acute rotenone exposure alters exploratory behavior, mobility, and Tyrosine hydroxylase (TH) expression.

(A–G) Open field: average speed (A), outer zone distance traveled (B), center/periphery distance ratio (C), freezing time (D), mobile time (E), immobile time (F), and mobility ratio (G).
(H–I) Elevated plus maze (EPM): total distance (H) and open arm distance (I).
(J–K) Tyrosine hydroxylase expression: TH mean fluorescence intensity (MFI) in the mid substantia nigra (SNc) (J) and in the substantia nigra reticulata (SNr) (K). Data are presented as mean ± SEM, with individual points representing single animals. Statistical analyses were performed using two-tailed Welch's *t*-tests. Sample sizes: open field, Control $n = 12$, Rot-treated $n = 15$; EPM, Control $n = 12$, Rot-treated $n = 16$; TH staining: Control n=5, Rot-treated n=7. Statistical significance is indicated as $P < 0.05$ (*), and $P < 0.0001$ (****).

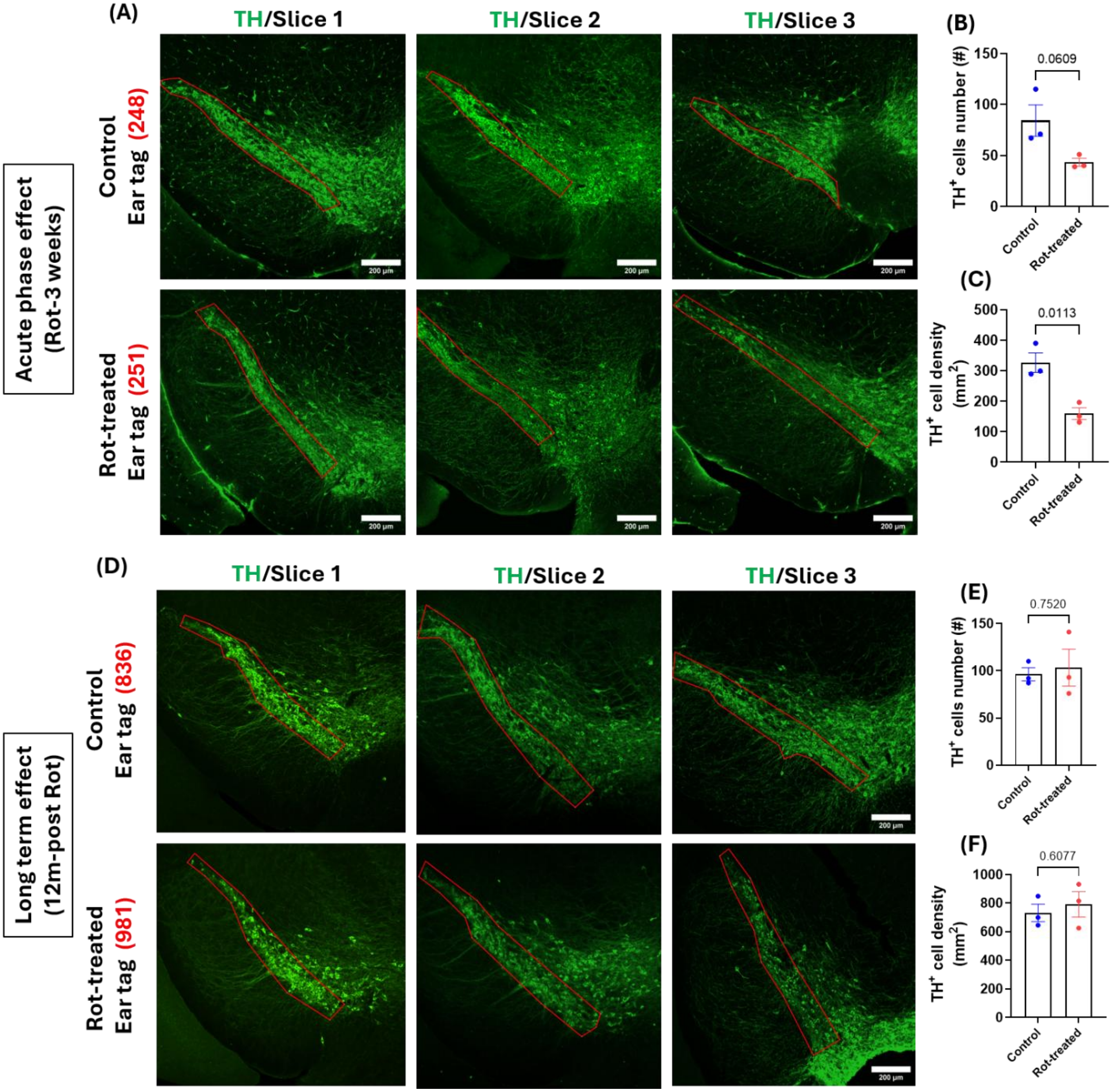

(A)
TH/Slice 1
TH/Slice 2
TH/Slice 3
Acute phase effect
(Rot-3 weeks)
Control
Ear tag (248)
Rot-treated
Ear tag (251)
200 µm
(B)
TH+ cells number (#)
0.0609
Control
Rot-treated
(C)
TH+ cell density
(mm2)
0.0113
(D)
TH/Slice 1
TH/Slice 2
TH/Slice 3
Long term effect
(12m-post Rot)
Control
Ear tag (836)
Rot-treated
Ear tag (981)
(E)
0.7520
(F)
0.6077

Figure S2. Stereological analysis of TH^{+} neuron number following acute rotenone exposure and its long-term effects.

Three brain sections from the same mice (control or Rot-treated) were analyzed for TH^{+} neuron counts. **(A, D)** Representative images of TH immunostaining (green) from the acute rotenone group (A) and the late stage (D). SNc area was measured within ROI (in red) **(B–C)** Quantification of the average number and density of TH^{+} neurons in SNc across three sections per mouse in the acute phase. **(E–F)** Quantification of the average number and density of TH^{+} neurons in SNc across three sections per mouse at the late stage. Data are presented as mean ± SEM. Statistical analyses were performed using two-tailed Welch's *t*-tests. Statistical significance: $P < 0.05$ (*), *ns = not significant. Scale bars, 200 μm, as indicated.*

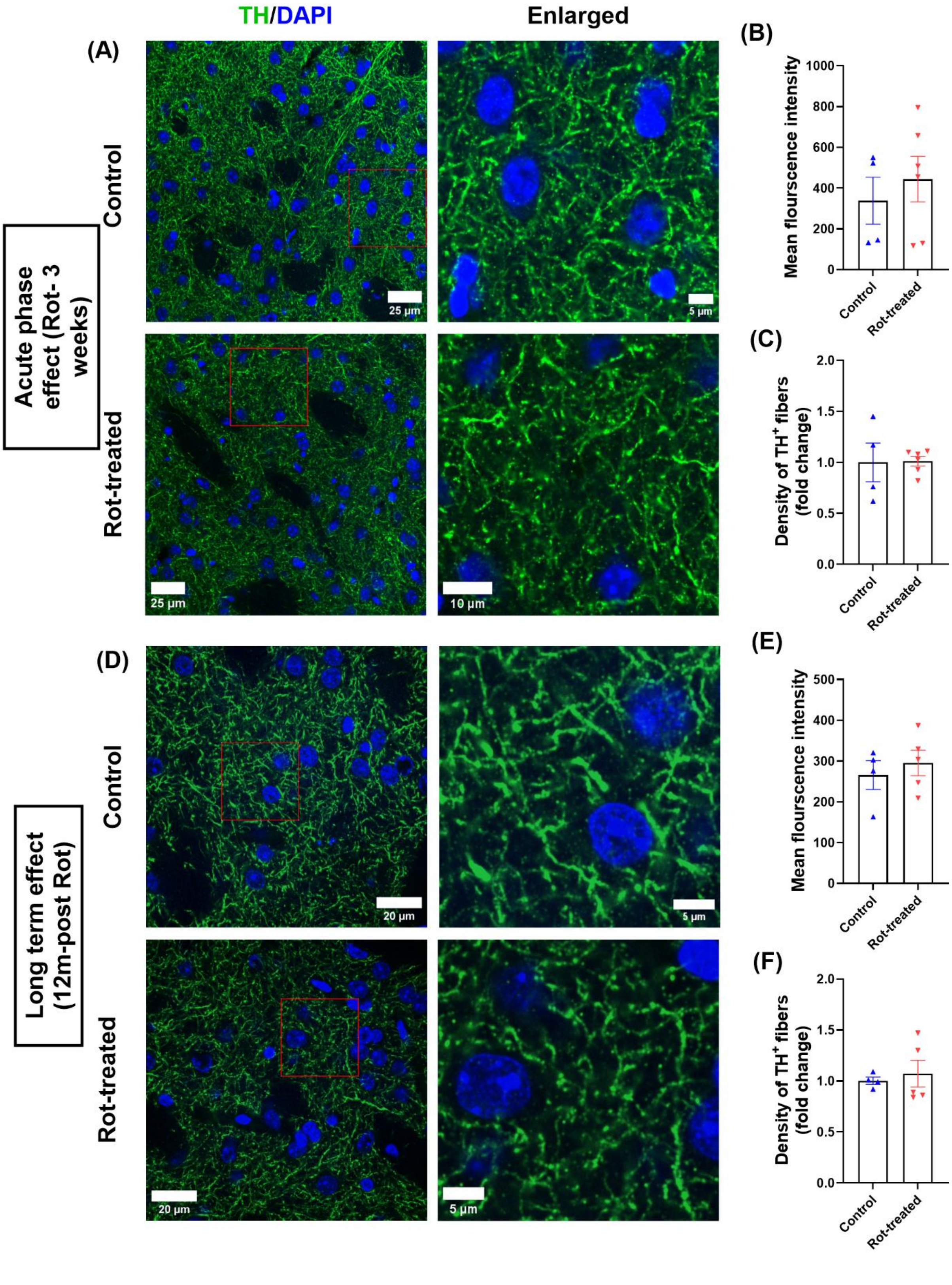
TH/DAPI
Enlarged
(A)
Acute phase effect (Rot- 3 weeks)
Control
Rot-treated
25 µm
5 µm
25 µm
10 µm
(B)
Mean flourscence intensity
Control
Rot-treated
(C)
Density of TH+ fibers (fold change)
Control
Rot-treated
(D)
Long term effect (12m-post Rot)
Control
Rot-treated
20 µm
5 µm
20 µm
5 µm
(E)
Mean flourscence intensity
Control
Rot-treated
(F)
Density of TH+ fibers (fold change)
Control
Rot-treated

Figure S3. Striatal TH was preserved after both short- and long-term rotenone exposure in mice.

(A) Representative confocal images of TH immunofluorescence (green) and nuclear counterstain (DAPI, blue) in the striatum after 3 weeks of rotenone exposure. The right-side panel shows enlarged views of individual TH^+ axons. **(B-C)** Intact TH fluorescence and density in the striatal dorsal lateral region at 1 month. **(D-F)** Like (A-C) but at 12 months. Data are presented as mean ± SEM, Mann-Whitney nonparametric test**;** Acute phase effect (Rot-3 weeks) (control n = 4, Rot-treated n = 6), Long term effect (12m-post Rot) (control n = 4, Rot-treated n = 5). Scale bars, 25 µm and 10 µm (panel A) and 20 µm and 5 µm (panel D).

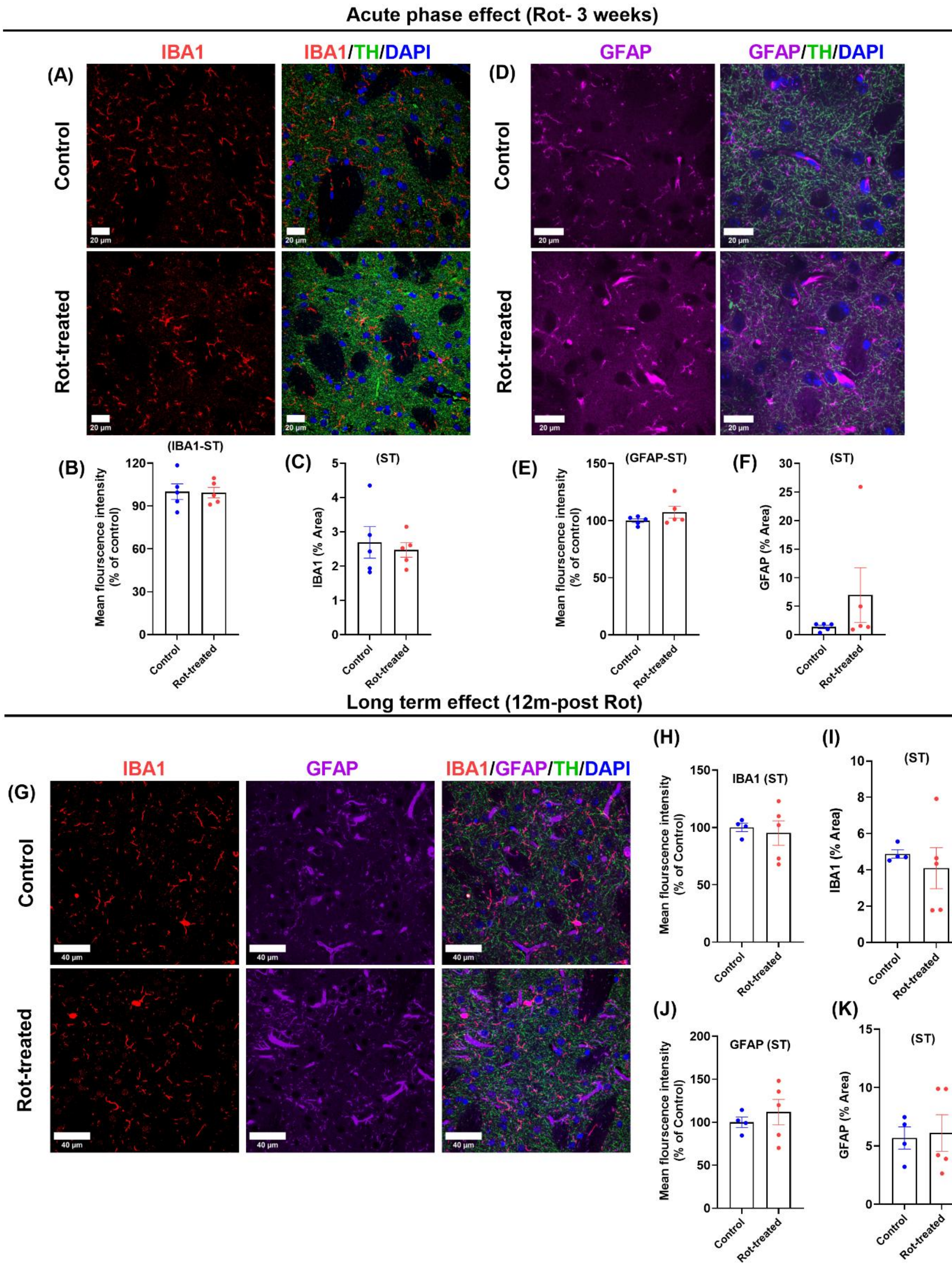

Acute phase effect (Rot- 3 weeks)
IBA1
IBA1/TH/DAPI
GFAP
GFAP/TH/DAPI
(A)
(D)
Control
Rot-treated
20 µm
(B)
(IBA1-ST)
Mean flourscence intensity (% of control)
(C)
(ST)
IBA1 (% Area)
(E)
(GFAP-ST)
Mean flourscence intensity (% of control)
(F)
(ST)
GFAP (% Area)
Control
Rot-treated
Long term effect (12m-post Rot)
(G)
IBA1
GFAP
IBA1/GFAP/TH/DAPI
40 µm
(H)
IBA1 (ST)
Mean flourscence intensity (% of Control)
(I)
(ST)
IBA1 (% Area)
(J)
GFAP (ST)
Mean flourscence intensity (% of Control)
(K)
(ST)
GFAP (% Area)

Figure S4. Rotenone exposure does not alter IBA1 and GFAP in the striatum.

(A) Representative confocal images of the striatum stained for IBA1 (red), tyrosine hydroxylase (green), GFAP (magenta), and DAPI (blue) at the acute phase (A, D) and at the late stage (G). **(B, C)** Quantification of Iba1 mean intensity (MFI) and IBA1-positive areas. **(E, F)** Quantification of GFAP mean intensity and GFAP-positive areas in the ST during the acute phase. **(H, I)** Quantification of Iba1 MFI and Iba1-positive areas. **(J, K)** Quantification of GFAP MFI and GFAP-positive areas in the ST at the late stage. Data are presented as mean ± SEM. Statistical analysis was performed using an unpaired two-tailed Welch's *t*-test. Sample sizes: Acute phase effect (Rot-3 weeks) (control n = 5, Rot-treated n = 5); Long term effect (12m-post Rot) (control n = 4, Rot-treated n = 5). Scale bars, 20 μm (panels A&D) and 40 μm (panel G).

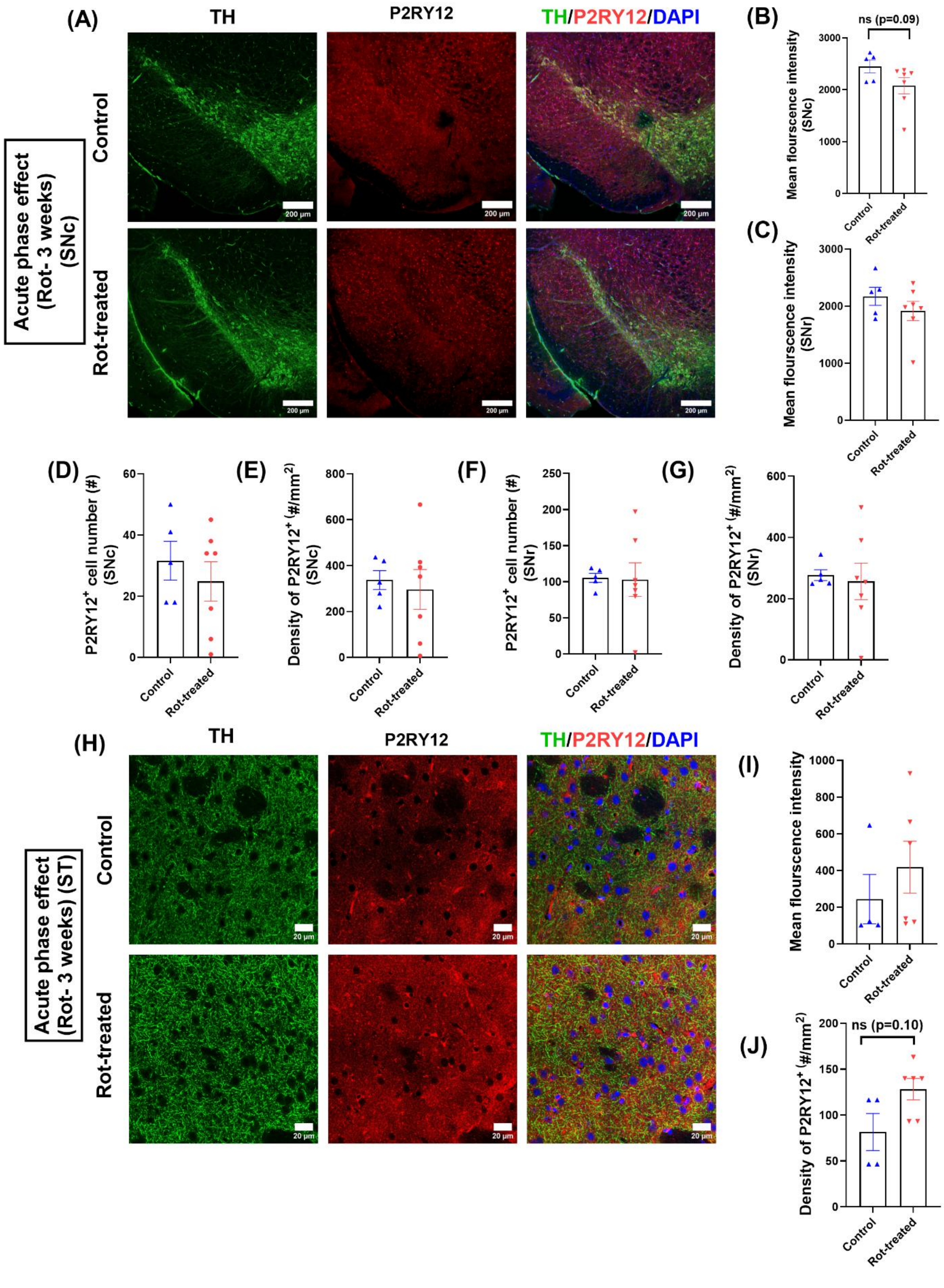
(A)
TH
P2RY12
TH/P2RY12/DAPI
Acute phase effect (Rot- 3 weeks) (SNc)
Control
Rot-treated
200 µm
(B)
ns (p=0.09)
Mean flourscence intensity (SNc)
(C)
Mean flourscence intensity (SNr)
(D)
P2RY12+ cell number (#) (SNc)
(E)
Density of P2RY12+ (#/mm2) (SNc)
(F)
P2RY12+ cell number (#) (SNr)
(G)
Density of P2RY12+ (#/mm2) (SNr)
(H)
Acute phase effect (Rot- 3 weeks) (ST)
20 µm
(I)
Mean flourscence intensity
(J)
ns (p=0.10)
Density of P2RY12+ (#/mm2)

Figure S5. Characterization of microglial P2RY12 changes in SNc and striatum at the acute phase of rotenone exposure.

(A) Representative confocal images of SNc showing TH (green), P2RY12 (red), and nuclei (DAPI; blue) from coronal sections of control and rotenone-treated mice after 3 weeks of rotenone treatment. **(B-C)** Microglial P2RY12 fluorescence intensity in the SNc and SNr. **(D-E)** P2RY12^{+} microglial cell number and cell density in the SNc, and (F-G) P2RY12^{+} microglial cell number and cell density in the SNr. (H) Confocal images of TH (green), P2RY12 (red), and nuclei (DAPI; blue) in the dorsal lateral striatum. **(I-J)** Intact P2RY12 fluorescence intensity and P2RY12^{+} cell density. Data are presented as mean ± SEM; Mann-Whitney nonparametric tests; SNC (control n = 5, Rot-treated n = 7), striatum (control n = 4, Rot-treated n = 6); ns, not significant. Scale bars, 200 µm (panel A) and 20 µm (panel H).

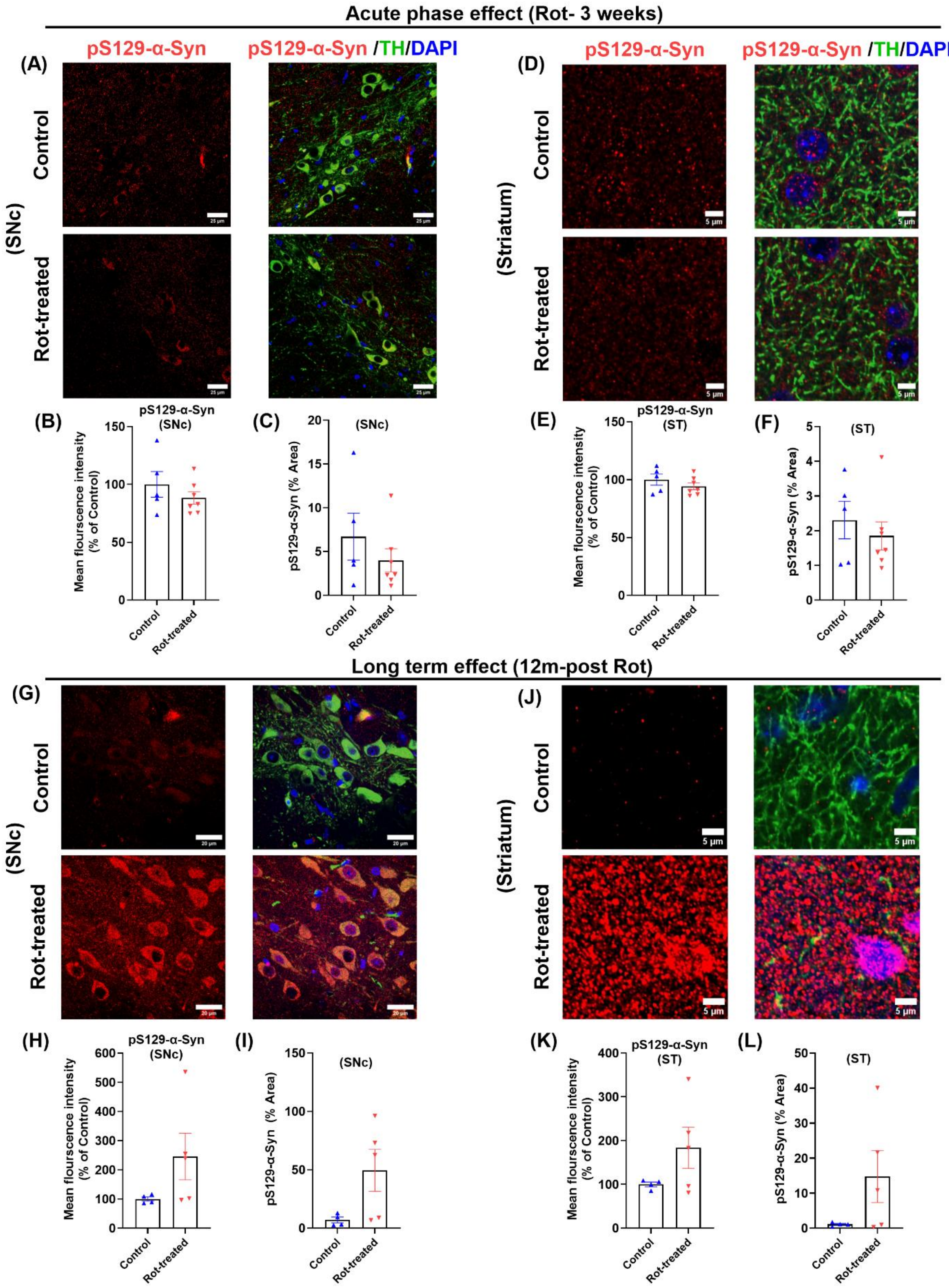
Acute phase effect (Rot- 3 weeks)
pS129-α-Syn
pS129-α-Syn /TH/DAPI
(A)
(D)
Control
Rot-treated
(SNc)
(Striatum)
(B) pS129-α-Syn (SNc)
Mean flourscence intensity (% of Control)
(C) (SNc)
pS129-α-Syn (% Area)
(E) pS129-α-Syn (ST)
(F) (ST)
Long term effect (12m-post Rot)
(G)
(J)
(H) pS129-α-Syn (SNc)
(I) (SNc)
pS129-α-Syn (% Area)
(K) pS129-α-Syn (ST)
(L) (ST)

Figure S6. Acute rotenone exposure and long-term effects do not significantly alter phosphorylated α-synuclein (pSer129 α-synuclein) aggregation in the substantia nigra and striatum.

(A) Representative confocal images of the SNc (A) and striatum (D) stained for phosphorylated α-synuclein (pSer129; red), tyrosine hydroxylase (TH; green), and DAPI (blue) from control and Rot-treated mice at the acute stage. **(B, C)** Quantification of pSer129 α-synuclein MFI and pSer129-positive area in the SNc. **(E, F)** Quantification of pSer129 MFI and pSer129-positive area in the ST during the acute phase. **(G, J)** Representative confocal images of SNc and ST stained for pSer129 α-synuclein from control and Rot-treated mice at the long-term stage. **(H, I)** Quantification of pSer129 MFI and pSer129-positive area in the SNc. **(K, L)** Quantification of pSer129 MFI and pSer129-positive area in the ST during the long-term stage. Data are presented as mean ± SEM. Statistical analysis was performed using unpaired two-tailed Welch's t-test or Mann–Whitney nonparametric test, as appropriate. Sample sizes: Acute phase effect (Rot-3 weeks) (control n = 5, Rot-treated n = 7); Long term effect (12m-post Rot) (control n = 4, Rot-treated n = 5). Scale bars, 25 μm and 5 μm (panels A&D) and 20 μm and 5 μm (panels G&J).

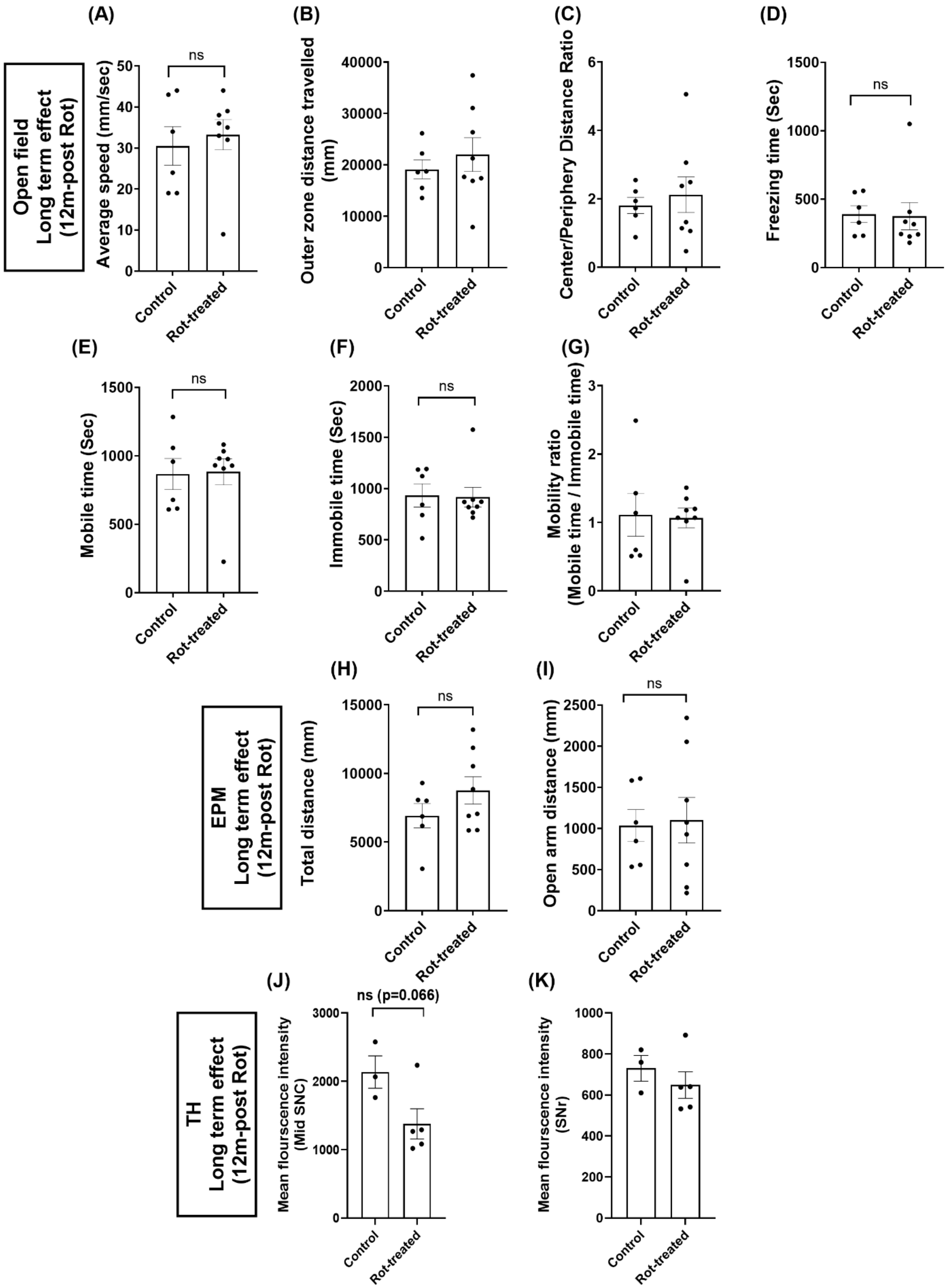

(A)
(B)
(C)
(D)
Open field
Long term effect
(12m-post Rot)
Average speed (mm/sec)
ns
Outer zone distance travelled (mm)
Center/Periphery Distance Ratio
Freezing time (Sec)
Control
Rot-treated
(E)
(F)
(G)
Mobile time (Sec)
Immobile time (Sec)
Mobility ratio
(Mobile time / Immobile time)
(H)
(I)
EPM
Long term effect
(12m-post Rot)
Total distance (mm)
Open arm distance (mm)
(J)
(K)
TH
Long term effect
(12m-post Rot)
ns (p=0.066)
Mean flourscence intensity
(Mid SNC)
Mean flourscence intensity
(SNr)

Figure S7. Long-term effects of rotenone exposure on exploratory behavior, mobility, and TH expression.

(A–G) Open field: average speed (A), outer zone distance traveled (B), center/periphery distance ratio (C), freezing time (D), mobile time (E), immobile time (F), and mobility ratio (G).
(H–I) Elevated plus maze (EPM): total distance (H) and open arm distance (I). **(J–K)** Tyrosine hydroxylase expression: TH mean fluorescence intensity (MFI) in the mid substantia nigra (SNc) (J) and in the substantia nigra reticulata (SNr) (K). Data are presented as mean ± SEM, with individual points representing single animals. Statistical analyses were performed using two-tailed Welch's *t*-tests. Sample sizes: open field, EPM, Control n = 6, Rot-treated n = 8; TH staining: Control n=3, Rot-treated n=5. Statistical significance, *ns = not significant.*

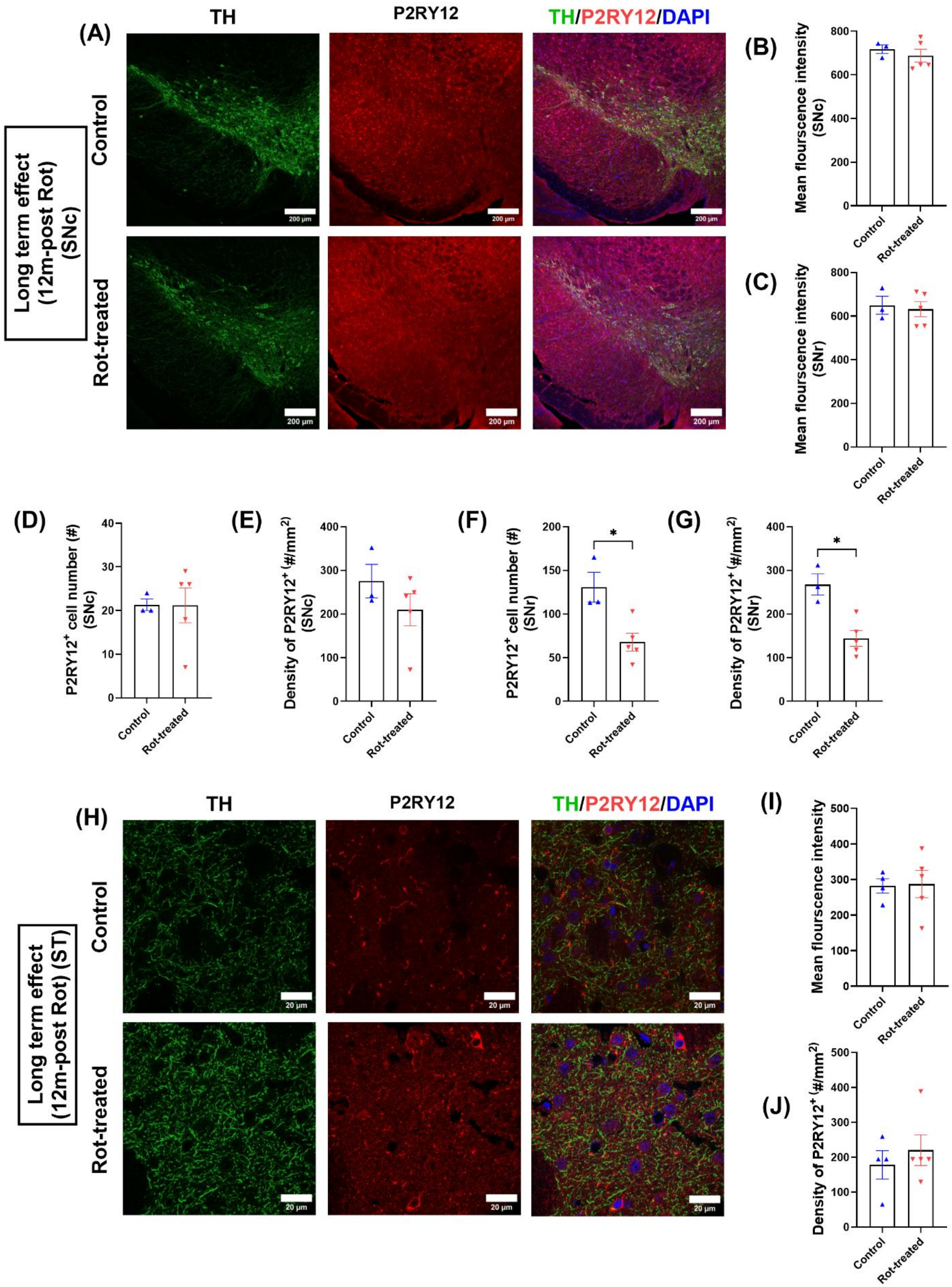
(A)
TH
P2RY12
TH/P2RY12/DAPI
Long term effect (12m-post Rot) (SNc)
Control
Rot-treated
200 µm
(B)
Mean flourscence intensity (SNc)
(C)
Mean flourscence intensity (SNr)
(D)
P2RY12+ cell number (#) (SNc)
(E)
Density of P2RY12+(#/mm2) (SNc)
(F)
P2RY12+ cell number (#) (SNr)
(G)
Density of P2RY12+(#/mm2) (SNr)
(H)
Long term effect (12m-post Rot) (ST)
20 µm
(I)
Mean flourscence intensity
(J)
Density of P2RY12+(#/mm2)
Control
Rot-treated

Figure S8. Microglial P2RY12 changes in the SNc and striatum at 12 months.

(A) Representative confocal images of SNc regions showing TH (green), P2RY12 (red), and nuclei (DAPI; blue) from control and rotenone-treated mice at 12 months. **(B-C)** Microglial P2RY12 fluorescence intensity in the SNc and SNr. **(D-E)** P2RY12^{+} microglial cell number and cell density in the SNc. **(F-G)** P2RY12^{+} microglial cell number and cell density in the SNr. (H) Confocal images of TH (green), P2RY12 (red), and nuclei (DAPI; blue) in the dorsal lateral striatum at 12 months. **(I-J)** Intact P2RY12 fluorescence intensity and P2RY12^{+} cell density. Data are presented as mean ± SEM; two-tailed Welch's *t* test; SNc (control n = 3, Rot-treated n = 5), striatum (control n = 4, Rot-treated n = 5); ns, not significant. Scale bars, 200 μm (panel A) and 20 μm (panel H).
